# Altered Behaviors and Impaired Synaptic Function in a Novel Rat Model With a Complete *Shank3* Deletion

**DOI:** 10.3389/fncel.2019.00111

**Published:** 2019-03-26

**Authors:** Tian-Jia Song, Xing-Yu Lan, Meng-Ping Wei, Fu-Jun Zhai, Tobias M. Boeckers, Jia-Nan Wang, Shuo Yuan, Meng-Ying Jin, Yu-Fei Xie, Wan-Wen Dang, Chen Zhang, Michael Schön, Pei-Wen Song, Mei-Hong Qiu, Ya-Yue Song, Song-Ping Han, Ji-Sheng Han, Rong Zhang

**Affiliations:** ^1^Department of Neurobiology, School of Basic Medical Sciences, Peking University, Beijing, China; ^2^Neuroscience Research Institute, Peking University, Beijing, China; ^3^Key laboratory for Neuroscience, Ministry of Education/National Health and Family Planning Commission, Peking University, Beijing, China; ^4^State Key Laboratory of Membrane Biology, School of Life Sciences, Peking University-IDG/McGovern Institute for Brain Research, Peking University, Beijing, China; ^5^Department of Neurobiology, Capital Medical University, Beijing, China; ^6^Institute for Anatomy and Cell Biology, Ulm University, Ulm, Germany; ^7^Department of Neurobiology, School of Basic Medical Sciences, Fudan University, Shanghai, China; ^8^School of Basic Medical Sciences, Tianjin Medical University, Tianjin, China; ^9^Wuxi HANS Health Medical Technology Co., Ltd., Wuxi, China

**Keywords:** *Shank3*, Phelan-McDermid syndrome, autism spectrum disorders, intellectual disabilities, social memory, pain threshold, oxytocin, vasopressin

## Abstract

Mutations within the *Shank3* gene, which encodes a key postsynaptic density (PSD) protein at glutamatergic synapses, contribute to the genetic etiology of defined autism spectrum disorders (ASDs), including Phelan-McDermid syndrome (PMS) and intellectual disabilities (ID). Although there are a series of genetic mouse models to study *Shank3* gene in ASDs, there are few rat models with species-specific advantages. In this study, we established and characterized a novel rat model with a deletion spanning exons 11–21 of *Shank3*, leading to a complete loss of the major SHANK3 isoforms. Synaptic function and plasticity of *Shank3*-deficient rats were impaired detected by biochemical and electrophysiological analyses. *Shank3*-depleted rats showed impaired social memory but not impaired social interaction behaviors. In addition, impaired learning and memory, increased anxiety-like behavior, increased mechanical pain threshold and decreased thermal sensation were observed in *Shank3*-deficient rats. It is worth to note that *Shank3*-deficient rats had nearly normal levels of the endogenous social neurohormones oxytocin (OXT) and arginine-vasopressin (AVP). This new rat model will help to further investigate the etiology and assess potential therapeutic target and strategy for *Shank3*-related neurodevelopmental disorders.

## Introduction

The *Shank3* gene is a member of the *Shank* gene family and encodes the SHANK3 protein, a key postsynaptic density (PSD) molecule at glutamatergic synapses (Naisbitt et al., 1999). SHANK3 is a scaffolding protein that interacts directly or indirectly with multiple glutamate receptors and the actin cytoskeleton critical for normal synaptic function and contains several specific protein domains, including an ANK (ankyrin repeats) domain, a SH3 (Src homology 3) domain, a PDZ (PSD95/Discs large/zona-occludens-1) domain, a proline-rich domain containing Homer binding regions and a SAM (sterile alpha motif) domain (Boeckers et al., 1999; Baron et al., 2006; Boeckers, 2006; Grabrucker et al., 2011).

Mutations within the *Shank3* gene are known to cause autism spectrum disorders (ASDs), which are characterized by impaired social interaction, communication skills and repetitive or restricted interests and behaviors. Haploinsufficiency of *Shank3* caused by a deletion or mutation accounts for up to 0.69% of autistic patients (Leblond et al., 2014). A specific form of a *Shank3*-dependent developmental disorder is Phelan-McDermid syndrome (PMS), which is also known as 22q13.3 deletion syndrome, with a deletion or single mutation in one copy of the *Shank3* gene (Wilson et al., 2003). PMS is characterized by intellectual disability (ID), developmental delays, absent or delayed speech, neonatal hypotonia and motor impairments, and autism or autistic-like behaviors are present in more than 80% of PMS patients (Soorya et al., 2013). Notably, many forms of ASDs present comorbidities, including ID (Polyak et al., 2015), attention deficits, hyperactivity (ADHD), anxiety (Croen et al., 2015), sensory-perceptual anomalies, pain insensitivity or self-injurious behaviors (Allely, 2013). In this respect, *Shank3* mutations play a key role in ID and cognitive impairments; for example, 1.7% of Chinese unexplained ID patients have 22q13 deletions (Gong et al., 2012). To further understand the etiology, the underlying neurobiological mechanisms and potential novel therapeutics, *Shank3*-related animal models need to be established and characterized.

Because of available and convenient genetic technologies, genetically modified mouse models are widely used despite several limitations of the species. To date, a series of mouse strains with *Shank3* deletions of different exons (Δe4–7, Δe4–9, Δe13–16, Δe9, Δe11, and Δe21) and a *Shank3* complete knockout mouse model have been established (Bozdagi et al., 2010; Bangash et al., 2011; Peca et al., 2011; Wang et al., 2011, 2016; Schmeisser et al., 2012; Yang et al., 2012; Kouser et al., 2013; Duffney et al., 2015; Lee et al., 2015). As expected, the *Shank3* mutation has been proven to affect synaptic proteins, synaptic morphology and related function in these *Shank3*-deficient mouse models. Most of these mice have been shown to have autistic-like behaviors, including impaired social and communication behaviors, increased repetitive behavior, and other phenotypes related to comorbidities, including anxiety behavior, deficits in learning and memory, impaired motor coordination ability and so on (Bozdagi et al., 2010; Bangash et al., 2011; Peca et al., 2011; Wang et al., 2011; Schmeisser et al., 2012; Yang et al., 2012; Kouser et al., 2013; Duffney et al., 2015; Lee et al., 2015; Wang et al., 2016). With some species-specific advantages compared to mouse models, genetically modified rat models make it possible to perform more complex and broader behavioral tests and to investigate related mechanisms in more precise brain regions of the larger brain. In addition, for pharmacological investigations and drug testing, rats are the prototypical animal model. However, to the best of our knowledge, only one *Shank3*-deficient rat model has been reported with impaired social memory and deficits in attention (Harony-Nicolas et al., 2017). In this study, a deletion was introduced in exon 6, which led to the disruption of the isoform-specific expression of SHANK3 (Harony-Nicolas et al., 2017). Interestingly, the *Shank3*-deficient rat model showed normal social interaction and self-grooming behaviors, which is different from those of *Shank3*-deficient mouse models. Therefore, more *Shank3* genetically modified rat models carrying different mutations need to be established and characterized, which may help to more comprehensively understand *Shank3* function, species-specific phenotype differences and the phenotypical diversity of clinical manifestations observed by clinicians and researchers; additionally, an animal model with a complete *Shank3* deletion can mimic the majority of patients carrying deletions of the entire *Shank3* gene in PMS (Soorya et al., 2013).

Oxytocin (OXT) and arginine-vasopressin (AVP) are two neuropeptides synthesized in the supraoptic nucleus (SON) and paraventricular nucleus (PVN) of the hypothalamus and are related to social interaction, social memory and anxiety in mammals (Harony and Wagner, 2010). Acute OXT treatment could improve impaired long-term social memory and nonsocial attention deficits in the *Shank3*-deficient rat model (Harony-Nicolas et al., 2017). Similar to the impact of OXT on this *Shank3*-deficient rats, the facilitation effect of OXT and AVP on social behavior has been observed in normal mice and rats (Lukas et al., 2011), low socially interacting rats (Zhang et al., 2015) and some genetic animal models (Jin et al., 2007; Gigliucci et al., 2014; Penagarikano et al., 2015). Interestingly, an impaired OXT/AVP system has been reported in autistic patients (Zhang et al., 2016) and some autistic animal models (Zhang et al., 2017); however, thus far, we know little about the endogenous OXT/AVP system in *Shank3*-deficient animal models.

In our present study, we established and characterized a novel rat model with a complete *Shank3* deletion, which adds to the existing *Shank3*-deficient rat model and can be compared with other *Shank3*-deficient animal models. It can also further help to understand the etiology and assess potential therapeutics of related neurodevelopmental disorders based on rat-specific advantages.

## Materials and Methods

### Generation of *Shank3* Knockout Rats

The *Shank3* knockout rat, in which the *Shank3* gene has been deleted from exon 11 to exon 21, was generated by the introduction of two sgRNAs with Cas9 to induce repair of the resultant two DSBs by NHEJ with a deletion of the intervening DNA sequence (Yang et al., 2013). Briefly, two sgRNAs were designed to target a region upstream of exon 11 or downstream of exon 21 of *Shank3*. For each targeting site, candidate guide RNAs were designed by the CRISPR design tool^1^ and screened for on-target activity. Then, the Cas9 mRNA and sgRNAs were transcribed *in vitro* using the MEGAshortscript T7 Transcription kit (AM1354, Invitrogen) and purified using the MEGAclear Transcription Clean-Up kit (AM1908, Invitrogen). Sprague-Dawley (SD) rat strains were used as embryo donors and pseudopregnant foster mothers. Superovulated female SD rats mated with SD stud males, and fertilized embryos were collected from the ampullae of the superovulated female SD rats. Different concentrations of Cas9 mRNA and sgRNAs were mixed and coinjected into the cytoplasm of one-cell stage fertilized eggs. After injection, surviving zygotes were transferred into oviducts of SD females to generate chimeras. Finally, positive SD founder rats mated to produce F1 heterozygous breeder pairs carrying approximately 26 kb deletions with the removal of the *Shank3* exon 11–21. The generation of *Shank3* knockout rats was conducted by Beijing Biocytogen Co. Ltd., Beijing, China.

Genotypes were determined by PCR of rat tail DNA using the primers F1 (CTGTTGGCTGAGCCTGGCATAGAG) and R1 (GCTGGAAAGAAACAACGAGAGCCAG) for the WT allele (559 base pairs) and the primers F2 (TTGTGCACTGCCTATGTTGACCACT) and R2 (TAGGCGAGAGAAGATGGTGTGATTTCC) for the mutant allele (688 base pairs).

### Animal Husbandry and Care

Male SD rats from heterozygous breeder pairs were used in all experiments. The rats were housed 3–5 per cage and maintained on a 12:12 h light/dark cycle (light on at 07:00 AM) with free access to food and water. All animal experimental procedures were approved by the Animal Care and Use Committee of Peking University (ethics approval ID, LA2015204) and in accordance with the U.S. National Institutes of Health Guide for the Care and Use of Laboratory Animals.

### Behavior Test

For all behavior tests, all genotypes were tested on the same day in a randomized order. Five- to six-week-old male rats were used for behavior testing unless otherwise stated. To minimize the impacts of repeated testing and handling, no more than four behavioral tests were performed in each cohort of rats.

#### Developmental Milestones

The rats were tested for developmental milestones from postnatal day (PND) 2 to 21. The parameters of physical developmental milestones included body weight, pinna detachment, incisor eruption and eye-opening. For each genotype, the number of pups that achieved these developmental goals were recorded.

#### Pup Separation-Induced Ultrasonic Vocalizations Test

A pup separation-induced ultrasonic vocalizations (USVs) test was performed on PND7 as previously described (Xu et al., 2015). Briefly, a pup was randomly chosen and was gently removed from the home cage and then transported to a clear plastic chamber (39 cm × 25 cm × 20 cm) on a heating pad (37°C) in a separate room. For each pup, the USVs were collected for 5 min by a condenser microphone (CM16/CMPA, Avisoft Bioacoustics, Germany) suspended approximately 25 cm above the base of the chamber with an amplifier (AUSG-116H, Avisoft Bioacoustics, Germany) set at a sampling rate of 250 kHz. After recording, the sound files were transferred to the sound analysis software SASLab Pro (Avisoft Bioacoustics, Germany) for fast Fourier transform with a 125 kHz low-pass filter. The classification algorithm of USVs was set as previously described (Li et al., 2011). The number and duration of each type of USVs were analyzed.

#### Juvenile Reciprocal Social Interaction Test

A juvenile reciprocal social interaction test was performed on PND 24–26 during the dark period of the light cycle under dim red illumination. Juvenile rats were habituated to the test arena (39 cm × 25 cm × 20 cm) for 15 min, and then an unfamiliar, age-matched, same-sex partner was introduced. The subject rat and the partner were allowed to move freely and videotaped for 10 min. The amount of time of total interactions was analyzed by a highly trained observer blinded to the genotype according to the parameters of social behaviors as previously described (Vanderschuren et al., 1995a,b).

#### Three-Chamber Test

A three-chamber test was performed on PND 35–42 during the dark period under dim red illumination. The apparatus using for testing was a rectangular, Plexiglas box divided into three chambers (40 cm × 34 cm × 24 cm), with the side chambers each connected to the middle chamber by a corridor (10 cm × 10 cm × 15 cm). The test consisted of three phases, named the adaptive phase, phase 1 and phase 2, according to previous studies with minor modifications (Zhang et al., 2015). During the adaptive phase, the subject rat was allowed to explore the entire apparatus freely for 5 min. During phase 1, an age- and sex-matched unfamiliar model rat was locked in a small cage made of stainless-steel wires as a social stimulus and then placed in one of the side chambers, and an identical but empty cage was placed in the other side chamber. The subject rat was allowed to explore the entire apparatus with the corridor opened and interact with the model rat for 10 min. During phase 2, another age- and sex-matched unfamiliar model rat was introduced to the empty cage as a novel social stimulus. The subject rat was also allowed to explore the entire apparatus and interact with the familiar and novel model rats for 10 min. The entire apparatus was cleaned with 75% ethanol after each trial concluded to eliminate the impact of residual rat odors. The amount of time spent in each of the three chambers was recorded automatically, and the rats were videotaped. The amount of time spent sniffing the wire cage was analyzed by a highly trained observer blinded to the genotype as closely social interaction time.

#### Self-Grooming Test

Repetitive behavior was analyzed by a self-grooming test during the dark period under dim red illumination in an empty cage (39 cm × 25 cm × 20 cm). The test consisted of a 10 min habituation and a followed 10 min test. The test session was videotaped, and the amount of time spent self-grooming was evaluated by a trained observer blinded to the genotype.

#### Olfactory Habituation/Dishabituation Test

Responses to nonsocial and social odors were analyzed by an olfactory habituation/dishabituation test (OHDT) during the dark period under dim red illumination as previously described with some modifications (Janssen et al., 2001; Li et al., 2015). The experiment was conducted in an acrylic cage (31 cm × 19 cm × 13 cm) with fresh bedding and a square metal gauze top. The test began with a 5 min familiarization followed by 15 sequential 2 min odor presentations with 1 min intervals. The odors consisted of three nonsocial odors, including water, almond (1:10 dilution, DJ-2372, Zhejiang Dahaojia Co., Ltd., Zhejiang, China) and vanilla (1:10 dilution, DJ-2301, Zhejiang Dahaojia Co., Ltd., Zhejiang, China) and two social odors, including social odor 1 (collected in the home cage consisting of three adult male rats without bedding) and social odor 2 (collected from adult female rats). Each odor, which was pipetted on a filter paper (70 mm diameter) placed on top of the gauze, was presented continuously three times. Sequences of three identical odor presentations evaluated habituation to the same smell. Switching to different odor presentations evaluated dishabituation to a new odor. The amount of time spent sniffing the odorized filter was quantitated by an observer blinded to the genotype.

#### Open Field Test

Anxiety behavior and spontaneous activity were evaluated in an acrylic box (100 cm × 100 cm × 40 cm) and videotaped by an overhead camera. Subject rats were initially placed in the center of the test arena and allowed to explore the arena freely for 10 min. Videos from each rat were processed using SMART software (v2.5.21, Panlab Harvard Apparatus). The amount of time spent in the peripheral zones of the open field was recorded as indices of anxiety-like behaviors. The total distances traveled in the open field were analyzed as indices of spontaneous activity.

#### Novel Object Recognition Test

Learning and memory were assessed by a novel object recognition test during the dark period under dim red illumination as performed in previous studies with some modifications (Ishikawa et al., 2014; Pyndt Jørgensen et al., 2015). Two days before the test, a subject rat was placed in the test arena (60 cm × 40 cm × 40 cm) for 10 min of habituation. During the training phase on the 3rd day, the rat was allowed to explore two identical objects in the arena for 20 min. After 1 h, during the test phase, in which one of the two objects was replaced by a new object (with a similar size but with different colors and shapes), the rat was introduced into the arena once again, allowed to explore freely for 10 min and videotaped. Then, the time spent exploring each of the two objects was measured by an observer blinded to the genotype. Object exploration behavior was defined as the nose of the rat touching the object or being oriented toward the object within 2 cm of it as previously described (Wang et al., 2011).

#### Von Frey Test

A von Frey test was used to assess the mechanical pain threshold of rats. Briefly, rats were restrained in transparent Lucite cubicles on an elevated metal mesh floor for 30 min to acclimate and then tested for the paw withdrawal threshold with calibrated von Frey filaments (bending forces ranging from 0.4 to 15 g) using the Dixon up-down method. The left hindpaw of a rat was stimulated perpendicularly to the central plantar surface, and withdrawal was determined if the rat lifted its paw. Finally, the withdrawal threshold was calculated and regarded as the index of the mechanical pain threshold.

#### Hot Plate Test

A hot plate test was used to detect the thermal pain threshold of rats. The apparatus consisted of a solid aluminum plate, which was heated and maintained at a constant temperature, confined by a transparent and removable Perspex cylinder. The temperature of the hot plate was set 53 ± 0.5°C. After a rat was placed on the hot plate surface, the amount of time for the rat to lick or lift its paw or jump off of the hot plate was defined as the paw withdrawal latency reflecting the temperature sensation. A cutoff time of 40 s was set to avoid tissue damage. The subject rat was tested three times with 15 min intervals and the mean of three recordings was reported as the final withdrawal latency.

#### Rotarod Test

Motor ability was analyzed by a rotarod test. Two days before the test, a subject rat was introduced into the apparatus for habituation (4 rpm). On the third day, during the test phase, the rat was introduced into the apparatus (4-40 rpm over 5 min) and was subjected to three trials with 15 min intervals. The latency to fall was recorded as the index of motor coordination and balance. The mean of three recordings was reported.

#### Hang Wire Test

A rat was placed midway on a horizontal metal gauze (1 mm diameter), which was held at a height of approximately 30 cm above the floor. After the subject rat crawled for a moment, the metal gauze was rotated 180 degrees, and the rat was allowed to hang. The latency to fall was recorded over 1 min; the test was performed three times per animal, and the results were averaged.

### Golgi Staining

Golgi staining was conducted in 5-week-old rats using a Hito Golgi-Cox OptimStainTM kit (HTKNS1125, Hitobiotec) according to the manufacturer’s instructions. Briefly, the brain was removed and transferred into a mixture of impregnation solution containing equal volumes of solution 1 and solution 2 at room temperature for 2 weeks in the dark. Then, brain tissue was transferred into solution 3 at 4°C for 24–72 h in the dark. Coronal sections (150 μm) were prepared with a freezing microtome (Leica-1950, Germany). The sections were stained using solutions 4 and 5 after mounting the sections onto the slides. Finally, the stained sections were imaged using a microscope (Olympus BX43, Tokyo, Japan) equipped with a 100× oil immersion lens. A researcher blinded to the genotype analyzed the spine density in the hippocampus using ImageJ (Wayne Rasband, National Institutes of Health, Bethesda, MD, USA). For each rat, nine different neurons were quantified from three slides. The spine density from three segments (70 μm) was averaged to provide a single value for each neuron.

### Electrophysiology

#### Slice Preparation

Hippocampal brain slices were prepared from 5- to 6-week-old male wild-type or *Shank3* knockout rats as previously described (Wei et al., 2016). Briefly, the rat brain was quickly removed and placed in ice-cold dissection solution (10 mM glucose, 213 mM sucrose, 1 mM NaH_2_PO_4_, 0.5 mM CaCl_2_, 5 mM MgCl_2_, 3 mM KCl and 26 mM NaHCO_3_, pH 7.4). Transverse slices were cut in ice-cold dissection solution on a vibrating blade microtome (Leica VT-1,200s, Wetzlar, Germany). The slices were moved to a chamber containing artificial cerebrospinal fluid (ACSF; 10 mM glucose, 2 mM NaH_2_PO_4_, 125 mM NaCl, 2.6 mM CaCl_2_, 1.3 mM MgCl_2_, 5 mM KCl, and 26 mM NaHCO_3_, pH 7.4, gassed with 95% O_2_ and 5% CO_2_) and incubated for 1 h at room temperature before recording.

#### Slice Electrophysiology

During recordings, slices were mounted on the stage of an upright microscope (Olympus BX51WI, Tokyo, Japan) and constantly perfused with ACSF using a gravity-driven perfusion system with a speed of 2 mL/min. The internal solution contained 145 mM KCl, 5 mM NaCl, 5 mM EGTA, 4 mM MgATP, 0.3 mM Na_2_GTP, 10 mM Hepes and 5 mM QX314 with the pH adjusted to 7.2 and an osmolarity of 305. Whole-cell recordings were conducted by adding 100 μM PTX (1128, Tocris) to ACSF (for the recording of evoked excitatory postsynaptic currents (EPSCs) mediated by AMPARs). The stimulus was delivered to Schaffer collaterals through a concentric bipolar electrode (CBBEB75, FHC, Bowdoin, ME, USA). For the recording of evoked EPSCs mediated by NMDARs, 100 μM PTX (1128, Tocris) and 10 μM CNQX (C127, Sigma) were added to ACSF, and the recordings were performed by holding the membrane potential at +40 mV. The stimulation intensity was 100 μA in evoked EPSCs recordings. Microelectrodes filled with ACSF (3–5 MΩ) were used to record field excitatory postsynaptic potentials (fEPSPs) from the stratum radiatum of the CA1 region. The stimulation intensity in fEPSPs recordings was defined as 60% of the maximum response. An EPC10 Patch Clamp Amplifier (HEKA, Lambrecht, Germany) was used to record the fEPSPs, and the values were calculated by measuring the onset (a 30–70% rising phase) slope of the fEPSP. For LTP recordings, we recorded a 20 min baseline followed by performing theta-burst stimulation [TBS; five episodes of stimulation delivered at 0.1 Hz, where each episode contained 10 stimuli trains of five pulses (100 Hz) delivered at 5 Hz] to induce LTP. Synaptic responses were collected every 15 s, and the data were analyzed by averaging four responses.

### Quantitative Immunoblot Analysis

#### Subcellular Fractionation

The brains of 6- to 8-week-old rats were removed quickly and separated into four regions, including the cortex, hippocampus, striatum and cerebellum. Brain tissues were homogenized with a motorized tissue grinder (G50, Coyote Bioscience, Campbell, CA, USA) at 3,000 rpm in buffer 1 [10 mM HEPES (pH 7.4), 2 mM EDTA, 5 mM sodium orthovanadate, 30 mM sodium fluoride, 20 mM β-glycerolphosphate, and protease inhibitor cocktail (Roche)]. The total homogenates were centrifuged at 500× *g* for 5 min at 4°C to remove nuclei, extracellular matrix and cell debris. The supernatant was collected and centrifuged at 10,000× *g* for 15 min at 4°C to separate the crude membrane fraction pellet 2 (P2). P2 was resuspended in 300–500 μl buffer 2 [50 mM HEPES (pH 7.4), 2 mM EDTA, 2 mM EGTA, 5 mM sodium orthovanadate, 30 mM sodium fluoride, 20 mM β-glycerolphosphate, 1% Triton-X-100, and protease inhibitor cocktail (Roche)] and centrifuged at 20,000× *g* for 80 min at 4°C to obtain pellet 3 (P3). P3 (Triton X-100 insoluble PSD fraction) was resuspended in 50 μl buffer 3 [50 mM Tris (pH 9), 5 mM sodium orthovanadate, 30 mM sodium fluoride, 20 mM β-glycerolphosphate, 1% NaDOC, and protease inhibitor cocktail (Roche)] and frozen in liquid nitrogen for storage at −80°C.

#### Western Blot Analysis

The total protein concentration was determined by Bradford assay using a Detergent Compatible Bradford Protein Assay kit (P0006C, Beyotime) according to the manufacturer’s instructions. For western blot analysis, equal amounts of 5–15 μg total homogenate (abbreviated to H_0_) and the PSD fraction (abbreviated to PSD) were loaded on the gels, transferred on a nitrocellulose membrane, and the membranes were blocked in 5% skim milk powder blocking solution using standard protocols. The membranes were subsequently incubated with primary antibodies on a horizontal shaker at 4°C overnight. After incubation, the membranes were further incubated with HRP-conjugated secondary antibodies on a horizontal shaker for 1 h at room temperature. Finally, the signals were visualized using an ECL Plus kit (PE0010, Solarbio). For quantification, the gray value of each band was analyzed by Quantity One software (Bio-Rad Laboratories, Irvine, CA, USA) and normalized to the gray value of β-actin. For SHANK proteins, different isoforms were all involved in the quantification. The following antibodies were used for western blot analysis: anti-SHANK1 (1:1,000 dilution, NB300–166, Neuromab), anti-SHANK2 (1:1,000 dilution, homemade from The Boeckers Lab, against amino acids 826–1259 of rat ProSAP1/SHANK2), anti-C-term SHANK3 (1:2,500 dilution, homemade from The Boeckers Lab, against both amino acids 781–1009 and amino acids 1260–1392 of rat ProSAP2/SHANK3), anti-N-term SHANK3 (1:500 dilution, homemade from The Boeckers Lab, against the first 120 N-terminal amino acids of rat SHANK3), anti-PSD-95 (1:500 dilution, AB2723, Abcam), anti-Homer1b/c (1:1,000 dilution, 160022, sysy), anti-GluR1 (1:1,000 dilution, AB1504, Millipore), anti-GluR2 (1:1,000 dilution, MAB397, Millipore), anti-NR1 (1:1,000 dilution, 556308BD, Biosciences), anti-NR2A (1:2,000 dilution, 07–632, Millipore), and anti-β-actin (1:1,000 dilution, TA-09, ZSGB-BIO).

### Brain Tissue Collection

For immunohistochemical analysis, 6- to 7-week-old rats were transcardially perfused with 0.9% saline solution and fixed with 4% paraformaldehyde solution (pH 7.2). Then, the brain was removed and postfixed in 4% paraformaldehyde solution (pH 7.2) overnight and cryoprotected in 15% sucrose solution at 4°C for 48 h followed by 30% sucrose solution for 5 days. Serial sections (30 μm each) were prepared using a freezing Microtome (Leica-1950, Germany) and stored at −20°C *in situ* hybridization protective solution.

For analysis of the mRNA levels of OXT, AVP and their receptors, the brain was quickly removed from 6- to 7-week-old rats and frozen in liquid nitrogen with embedding medium. According to the Paxinos and Watson Rat Brain Atlas, bilateral micropunches (1 mm in diameter for PVN and 1.5 mm for others) were obtained from the following regions using a freezing Microtome (Leica-1950, Germany): the PVN and SON of the hypothalamus, basolateral nucleus of the amygdala (BLA), central nucleus of the amygdala (CeA), medial nucleus of the amygdala (MeA) and the lateral septum [LS; (PVN: Bregma −0.60 to −1.92 mm, SON: Bregma −0.48 to −1.72 mm, BLA: Bregma −1.72 to −3.60 mm, CeA: Bregma −1.72 to −3.00 mm, MeA: Bregma −1.72 to −3.60 mm, and LS: Bregma 0.96 to −0.12 mm)].

### Immunohistochemistry

To estimate OXT- and AVP-immunoreactive cells in the PVN and SON, target sections were chosen and preincubated in 0.3% H_2_O_2_ for 15 min at 37°C to eliminate endogenous peroxidase activity. After preincubation in 0.3% H_2_O_2_, floating sections were incubated in blocking buffer containing 10% goat serum and 0.3% Triton X-100 in 0.01 M PBS (pH 7.2) for 60 min at 37°C and then incubated with specific primary antibodies at 37°C for 2 h followed by 4°C for 24–48 h. The following primary antibodies were used: anti-OXT (1:2,000 dilution, AB2078, Abcam) and anti-AVP (1:2,000 dilution, AB39363, Abcam). For OXT- and AVP-reactive cells determination, the sections were incubated with the enzyme-labeled secondary antibody from the DAB staining kit (PV-6001, ZSGB-BIO) at room temperature for 1 h and then stained by the DAB staining method using this kit. Finally, the floating sections were attached to a glass slide under a coverslip with mounting medium and imaged using a microscope (Leica DMI400B, Wetzlar, Germany) with a camera for counting. OXT- and AVP-immunoreactive cells were identified as cells containing brown immunoreactive deposits (OXT and AVP) in the nuclei. The total number of OXT- and AVP-immunoreactive cells were counted unilaterally in the PVN and SON from six sections between Bregma −0.8 and −2.1 mm by researchers blinded to the genotype using ImageJ (Wayne Rasband, National Institutes of Health, Bethesda, MD, USA).

### Real-Time Quantitative PCR

Total mRNA was extracted from the brain tissue micropunches using TRIzol reagent (Invitrogen, Carlsbad, CA, USA) according to the manufacturer’s instructions. The RNA sample was digested with DNase (Promega, Madison, WI, USA) to remove DNA contamination. Then, cDNA was synthesized from the sample using a PrimeScript RT-PCR kit (TaKaRa, Dalian, China). Expression levels of the target genes (OXT, AVP, OXTR, and V1aR) and the endogenous control gene (β-actin) were analyzed by real-time quantitative PCR using TaqMan^®^ Gene Expression Assays (assay ID: OXT—Rn00564446_g1, AVP—Rn00690189_g1, OXTR—Rn00563503_m1, V1aR—Rn00583910_m1, β-actin—Rn00667869_m1). The RT PCR procedure was performed in an optical 96-well reaction plate in duplicate on a 7500 RT PCR System (Applied Biosystems, Foster City, CA, USA) under standard amplification conditions as follows: 2 min at 50°C, 10 min at 95°C, 40 cycles of 15 s at 95°C and 1 min at 60°C. Finally, the data were transformed using the ΔΔCT method with β-actin as the reference gene and normalized to the wild-type samples for comparison.

#### Statistics

IBM SPSS Statistics 19 (SPSS Inc., Chicago, IL, USA) and GraphPad Prism 5.0 (GraphPad Software Inc., San Diego, CA, USA) was used for statistical analyses and generating graphs. For the comparisons, parametric tests including *t*-tests and one-way analysis of variance (ANOVA) were used if the data were normally distributed (distribution tested by the Shapiro-Wilk normality test), and nonparametric approaches, including the Wilcoxon test and Kruskal-Wallis test, were used for data with a nonnormal distribution. Pearson’s chi-squared test was used to assess rank variables. For all data, the results were expressed as the mean ± standard error of the mean (SEM), and *P* < 0.05 (two-tailed) was considered statistically significant.

## Results

### Generation of *Shank3*-Deficient Rats

**Figure 1:**
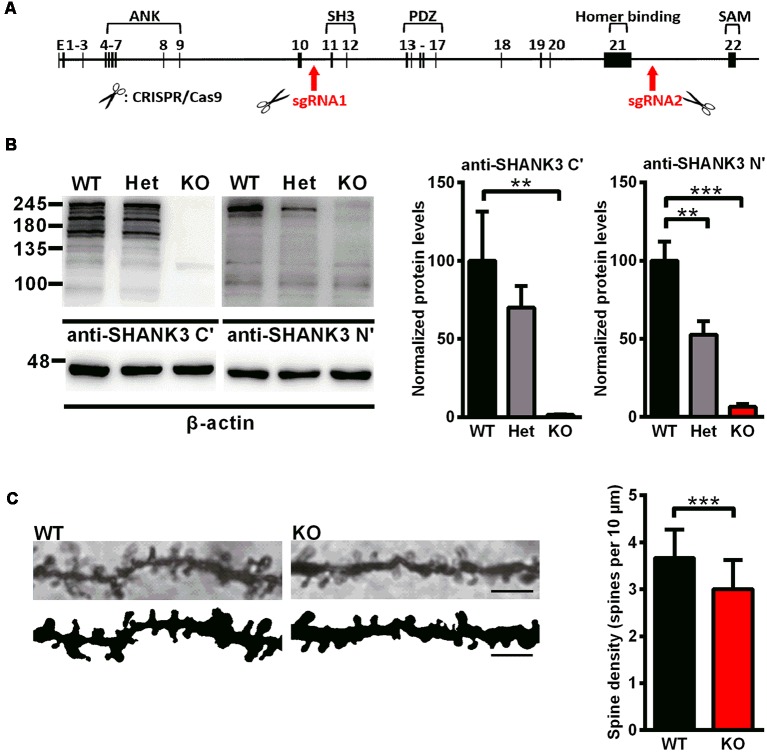
Generation of *Shank3*-deficient rats and reduced spine density in *Shank3* knockout rats. **(A)** Targeting strategy for the *Shank3* gene. The top schematic shows the genomic structure of the *Shank3* gene. The arrows indicate the targeting site of the CRISPR/Cas9 system. **(B)** Representative immunoblots of SHANK3 in hippocampal postsynaptic density (PSD) fractions probed with two anti-SHANK3 antibodies directed against the C-terminus or N-terminus (left panel). Quantification of the SHANK3 expression levels normalized to β-actin [right panel; WT, *n* = 5; Het, *n* = 5–6; KO, *n* = 6; one-way analysis of variance (ANOVA) with Dunnett’s multiple comparisons test]. **(C)** Representative images of dendritic spines from hippocampal neurons (scale bar: 10 μm; left panel) and quantification of spine density (right panel; *n* = 3 rats for each genotype, nine neurons per rat; unpaired *t*-test). Data are presented as the mean ± standard error of the mean (SEM). **P < 0.01; ***P < 0.001.

*Shank3*-deficient rats were generated using the CRISPR/Cas9 approach. As shown in the genomic structure of *Shank3*, two sgRNAs were designed to target the upstream region of exon 11 and the downstream region of exon 21 in the *Shank3* gene (Figure 1A). The region spanning these two targeting sites (approximately 26 kb) were deleted, including the SH3 domain, the PDZ domain and the proline-rich region. All subject rats used in the experiments were from heterozygous breeder pairs. The offspring genotypes were identified by PCR with rat tail DNA. For the WT allele, the PCR product was 559 base pairs, and for the mutant allele, the PCR product was 688 base pairs.

To analyze the expression of the SHANK3 protein, western blotting was performed with an antibody targeted against the proline-rich domain of SHANK3. The results showed that there were not any SHANK3 isoforms in *Shank3* knockout rats and low expression levels (approximately 64%) in heterozygous rats (Figure 1B). Another antibody targeted against the N-terminal amino acids of SHANK3 was also used, and the results showed that the full-length SHANK3 protein was completely absent in *Shank3* knockout rats and expressed to a lesser extent (approximately 53%) in heterozygous rats (Figure 1B). To confirm the expression of the SHANK3 protein in different brain regions, total homogenate (H_0_) and isolated PSD fractions were all analyzed from the hippocampus, cortex, striatum and cerebellum. The results showed that there was no expression of full-length SHANK3 protein in all samples from the hippocampus, cortex and striatum of *Shank3* knockout rats and a reduced expression level in samples of heterozygous rats (Supplementary Figure S1). For the short isoform of SHANK3 in the cerebellum, there was also no expression in *Shank3* knockout rats (Supplementary Figure S1).

### Reduced Spine Density in *Shank3*-Deficient Rats

Over 90% of excitatory synapses in the brain are located on dendritic spines (Gray, 1959), which undergo dramatic changes during development. We analyzed the dendritic spine density in the hippocampus by Golgi staining on PND35, at the time that spinogenesis was nearly completed (Bian et al., 2015). In *Shank3* knockout rats, the spine density was significantly decreased compared to that of the wild-type littermates (*Shank3*^+/+^: 3.668 ± 0.1161/10 μm; *Shank3*^−/−^: 3.006 ± 0.1191/10 μm; *n* = 9 neurons from three rats per genotype, *P* < 0.001; Figure 1C), which indicated impaired spinogenesis in the hippocampus of the *Shank3* knockout rats.

### Altered Synaptic Proteins in *Shank3*-Deficient Rats

**Figure 2:**
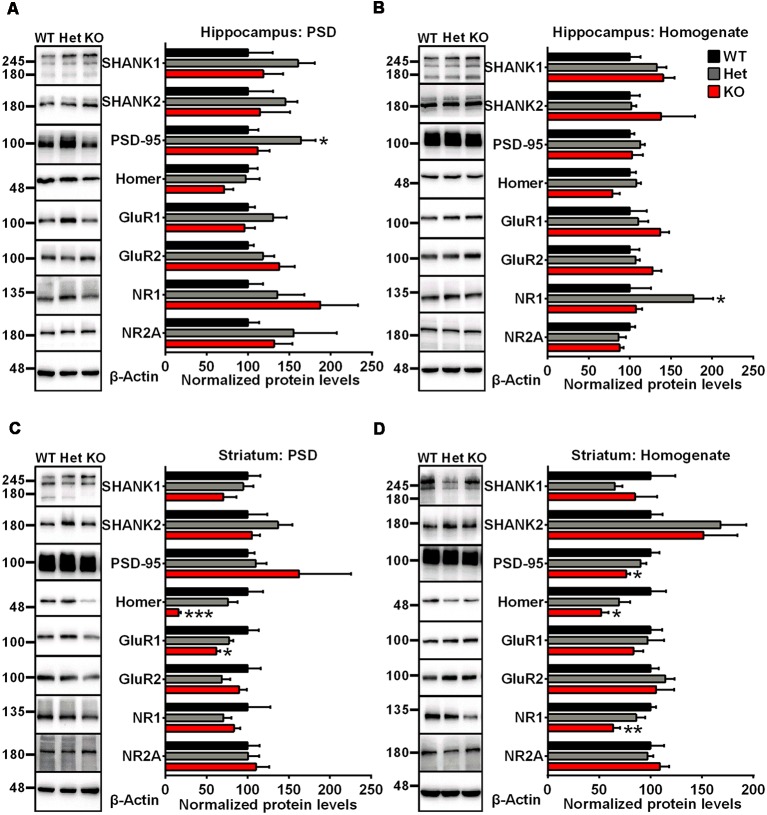
Altered synaptic proteins in PSD fractions and total homogenates from the hippocampus and striatum of *Shank3*-deficient rats. **(A)** Representative immunoblots of PSD fractions from the hippocampus probed with different antibodies as indicated (left panel). Quantification of the corresponding protein expression levels normalized to β-actin (right panel). **(B)** Representative immunoblots of total homogenates from the hippocampus probed with different antibodies as indicated (left panel). Quantification of the corresponding protein expression levels normalized to β-actin (right panel). **(C)** Representative immunoblots of PSD fractions from the striatum probed with different antibodies as indicated (left panel). Quantification of the corresponding protein expression levels normalized to β-actin (right panel). **(D)** Representative immunoblots of total homogenates from the striatum probed with different antibodies as indicated (left panel). Quantification of the corresponding protein expression levels normalized to β-actin (right panel; WT, *n* = 5; Het, *n* = 5–6; KO, *n* = 6; one-way ANOVA with Dunnett’s multiple comparisons test). Data are presented as the mean ± SEM. *P < 0.05; **P < 0.01; ***P < 0.001.

To examine synaptic protein alterations in *Shank3*-deficient rats, we performed quantitative immunoblot analysis for a series of proteins interacting with SHANK3 at the PSD, including SHANK family proteins (SHANK1 and SHANK2), scaffolding proteins (PSD-95 and Homer) and several glutamate receptor subunits (GluR1, GluR2, NR1 and NR2A). Total homogenate (H_0_) and isolated PSD fractions were analyzed from the hippocampus, striatum, cortex and cerebellum of 6- to 8-week-old rats. In PSD fractions, the expression levels of Homer and GluR1 were significantly lower in the striatum of *Shank3* knockout rats compared to those in the wild-type controls (16.02 ± 2.864% of *Shank3*^+/+^ for Homer, *P* < 0.001; 62.01 ± 4.369% of *Shank3*^+/+^ for GluR1, *P* < 0.05; *n* = 5–6 per genotype; Figure 2C). The expression levels of PSD-95, Homer and NR1 were also significantly reduced in homogenates from the striatum of *Shank3* knockout rats compared with wild-type control samples (76.27 ± 3.863% of *Shank3*^+/+^ for PSD-95, *P* < 0.05; 51.86 ± 7.439% of *Shank3*^+/+^ for Homer, *P* < 0.05; 63.73 ± 6.608% of *Shank3*^+/+^ for NR1, *P* < 0.01; *n* = 5–6 per genotype; Figure 2D). We observed that the expression level of PSD-95 was significantly increased in the hippocampal PSD fraction of heterozygous rats (164.2 ± 17.68% of *Shank3*^+/+^, *P* < 0.05; *n* = 5–6 per genotype; Figure 2A), and the expression level of NR1 significantly increased in the hippocampal homogenate fraction of heterozygous rats (177.4 ± 23.63% of *Shank3*^+/+^, *P* < 0.05; *n* = 5–6 per genotype; Figure 2B). The expression level of NR1 was significantly reduced in PSDs from the cerebellum of heterozygous rats (59.59 ± 8.231% of *Shank3*^+/+^, *P* < 0.05; *n* = 5–6 per genotype; Supplementary Figure S2C). No genotype differences were observed for synaptic proteins in homogenate and PSDs from the cortex and homogenate from the cerebellum (Supplementary Figures S2A,B,D). These results above indicated altered synaptic proteins in *Shank3*-deficient rats.

### Impaired Synaptic Function and Plasticity in *Shank3*-Deficient Rats

**Figure 3:**
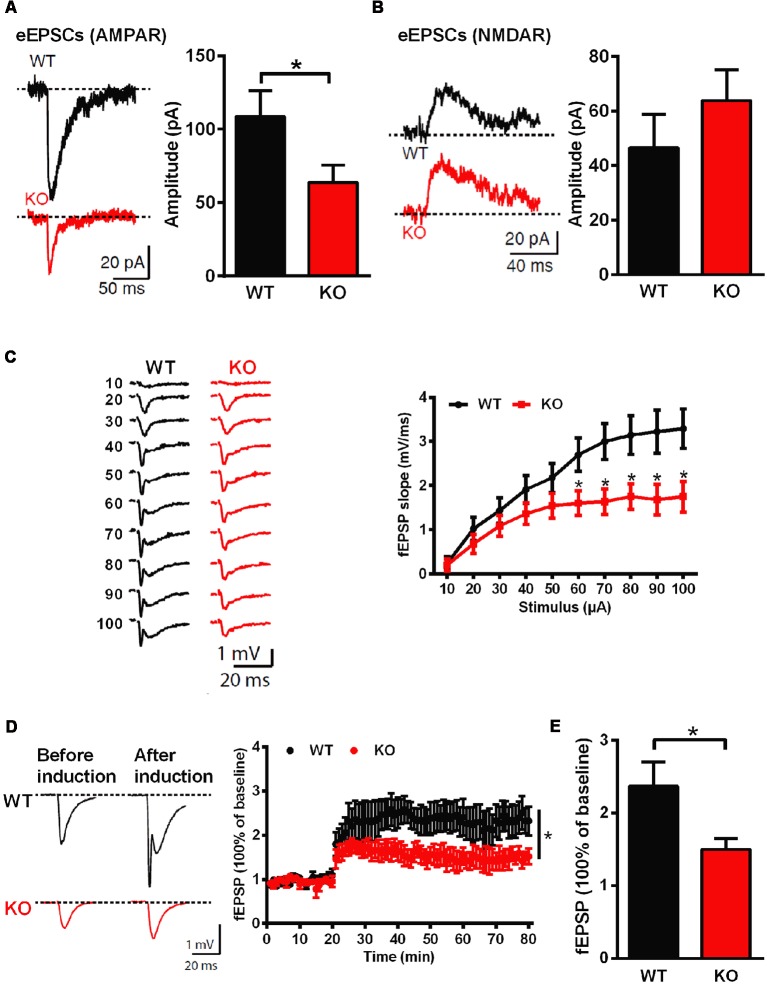
Impaired hippocampal glutamatergic synaptic transmission and long-term potentiation (LTP) in *Shank3*-deficient rats. **(A)** Evoked excitatory postsynaptic currents (EPSCs) mediated by AMPAR in CA1 pyramidal cells. The sample traces of individual recordings (left panel) and the average amplitude of all eEPSCs (right panel; WT, *n* = 16 neurons from three rats; KO, *n* = 17 neurons from three rats; unpaired *t*-test). **(B)** Evoked EPSCs mediated by NMDAR in CA1 pyramidal cells. The sample traces of individual recordings (left panel) and the average amplitude of all eEPSCs (right panel; WT, *n* = 23 neurons from four rats; KO, *n* = 29 neurons from four rats; unpaired *t*-test). **(C)** Input-output curves for basal synaptic transmission in CA1. The sample traces of individual recordings (left panel) and the average fEPSP slope (right panel; WT, *n* = 6 slices from three rats; KO, *n* = 7 slices from three rats; unpaired *t*-test). **(D)** LTP induced by theta-burst stimulation (TBS) in CA1. The sample traces before and after LTP induction (left panel) and the average time plot (right panel; WT, *n* = 6 slices from three rats; KO, *n* = 8 slices from three rats; two-way ANOVA). **(E)** The mean fEPSP slope averaged 50–60 min after LTP induction (WT, *n* = 6 slices from three rats; KO, *n* = 8 slices from three rats; unpaired *t*-test). Data are presented as the mean ± SEM. *P < 0.05.

Whole-cell patch clamp and extracellular field recordings in acute hippocampal slices were recruited to analyze the influence of *Shank3* deficiency on synaptic function and plasticity. The amplitude of AMPARs-mediated but not NMDARs-mediated evoked EPSCs was significantly reduced in *Shank3* knockout rats (*Shank3*^+/+^: 108.6 ± 17.55 pA, *n* = 16 neurons from three rats; *Shank3*^−/−^: 63.74 ± 11.84 pA, *n* = 17 neurons from three rats; *P* < 0.05; Figures 3A,B). Furthermore, we analyzed fEPSP in the CA1 region, and the input-output curves demonstrated a significantly decreased fEPSP slope in *Shank3* knockout rats (*Shank3*^+/+^: *n* = 6 slices from three rats; *Shank3*^−/−^: *n* = 7 slices from three rats; *P* < 0.05; Figure 3C). In addition to deficits in basal synaptic transmission, long-term potentiation (LTP) induced by theta-burst stimulation (TBS) was also reduced in *Shank3* knockout rats (last 10 min: *Shank3*^+/+^: 237.1 ± 33.50% of baseline, *n* = 6 slices from three rats; *Shank3*^−/−^: 150.0 ± 14.97% of baseline, *n* = 8 slices from three rats; *P* < 0.05; Figures 3D,E). These results above showed impaired synaptic function and plasticity in *Shank3*-deficient rats.

### Normal Social Interaction Behavior but Impaired Social Memory and Impaired Learning and Memory in *Shank3*-Deficient Rats

A series of behavioral tests were performed to determine the phenotype of *Shank3*-deficient rats. To determine whether the *Shank3* mutation impacts basic developmental processes in rats, developmental milestones were evaluated, including body weight, pinna detachment, incisor eruption and eye-opening. There were no significant genotype differences in the above-mentioned measures or general health (Supplementary Figure S3). We also observed no genotype differences in pup separation-induced USVs on PND7 (data not shown) and reciprocal social interaction behaviors (data not shown).

**Figure 4:**
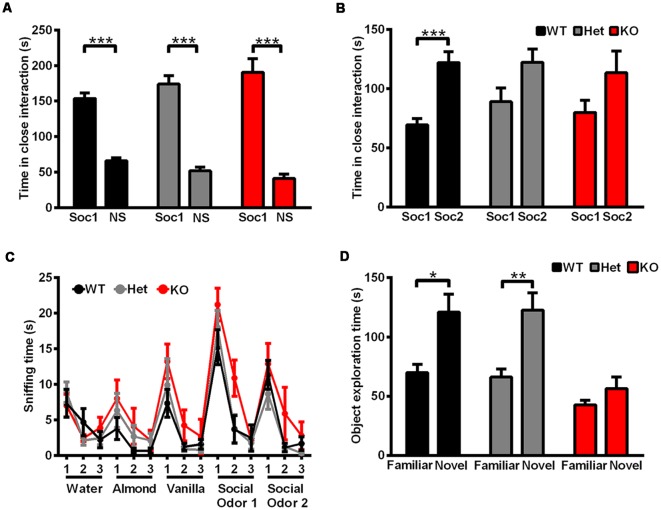
*Shank3*-deficient rats exhibited normal social interaction behavior but impaired social memory and impaired learning and memory.** (A)** The amount of time spent investigating either the social (Soc1) or nonsocial (NS) stimulus in phase 1 of the three-chamber test (WT, *n* = 19, paired *t*-test; Het, *n* = 24, paired *t*-test; KO, *n* = 10, paired *t*-test). **(B)** The amount of time spent investigating either the familiar social (Soc1) or novel social (Soc2) stimulus in phase 2 of the three-chamber test (WT, *n* = 19, paired *t*-test; Het, *n* = 24, paired *t*-test; KO, *n* = 10, paired *t*-test). **(C)** The amount of time spent sniffing a sequence of identical or novel odors in the olfactory habituation/dishabituation test (OHDT; *n* = 9 rats for each genotype). **(D)** The amount of time spent investigating either the familiar or novel object in the novel object recognition test (WT, *n* = 10, Wilcoxon test; Het, *n* = 18, Wilcoxon test; KO, *n* = 17, Wilcoxon test). Data are presented as the mean ± SEM. *P < 0.05; **P < 0.01; ***P < 0.001.

Social interaction behaviors and social memory were examined by a three-chamber test. In phase 1 of the three chamber test, when the subject rat was exposed to a social stimulus and a nonsocial stimulus simultaneously, *Shank3*-deficient and wild-type rats both spent more time in the compartment containing the social stimulus (data not shown) or in close interaction with the social partner (Figure 4A), which showed normal social interaction behaviors and social preferences in *Shank3*-deficient rats. In phase 2 of the three chamber test, a novel social partner was introduced, when the subject rat was exposed to a social and a novel social stimulus simultaneously. Wild-type rats showed a preference for the novel social partner, which was indicated by the increase in the amount of time spent in the compartment containing the novel social stimulus (data not shown) and the amount of time spent in close interaction with the novel social partner (69.42 ± 5.134 s spent in close interaction with the familiar social partner and 122.1 ± 9.124 s spent in close interaction with the novel social partner, *n* = 19, *P* < 0.0001; Figure 4B). However, *Shank3*-deficient rats did not show social novelty recognition, as indicated by a similar amount of time spent in the two compartments (data not shown) and the similar amount of time spent in close interaction with the two social partners (*Shank3*^+/–^: 88.83 ± 11.79 and 122.2 ± 11.18 s, respectively, *n* = 24, *P* = 0.1006; *Shank3*^−/−^: 79.90 ± 10.09 and 113.4 ± 18.33 s, respectively, *n* = 10, *P* = 0.2157; Figure 4B), indicating impaired social memory in *Shank3*-deficient rats.

Since olfactory ability is important for the social memory of rodents (Wacker and Ludwig, 2012), olfactory habituation/dishabituation was tested in three genotype groups. For nonsocial and social odor cues, both *Shank3*-deficient and wild-type rats showed olfactory habituation and dishabituation, as indicated by the decreased amount of time spent in sniffing the three sequences of the same odors and increased amount of time spent in sniffing the different odors (Figure 4C). Therefore, the deficits in social novelty recognition of *Shank3*-deficient rats may not be due to olfactory ability.

Cognitive performance was examined by a novel object recognition test. After a novel object was introduced to replace one of the familiar objects, wild-type and heterozygous rats showed preference for the novel object, which was indicated by the increased amount of time spent exploring the novel object (*Shank3*^+/+^: 69.8 ± 6.955 s spent exploring the familiar object and 120.9 ± 15.19 s spent exploring the novel object, *n* = 10, *P* < 0.05; *Shank3*^+/–^: 66.06 ± 6.976 s spent exploring the familiar object and 122.7 ± 14.55 s spent exploring the novel object, *n* = 18, *P* < 0.01), while *Shank3* knockout rats showed no preference (*Shank3*^−/−^: 42.65 ± 3.918 and 56.35 ± 9.823 s, respectively, *n* = 17, *P* = 0.3529; Figure 4D), which suggested impaired learning and memory in *Shank3* knockout rats.

### Increased Anxiety Behavior and Pain Threshold in *Shank3*-Deficient Rats

**Figure 5:**
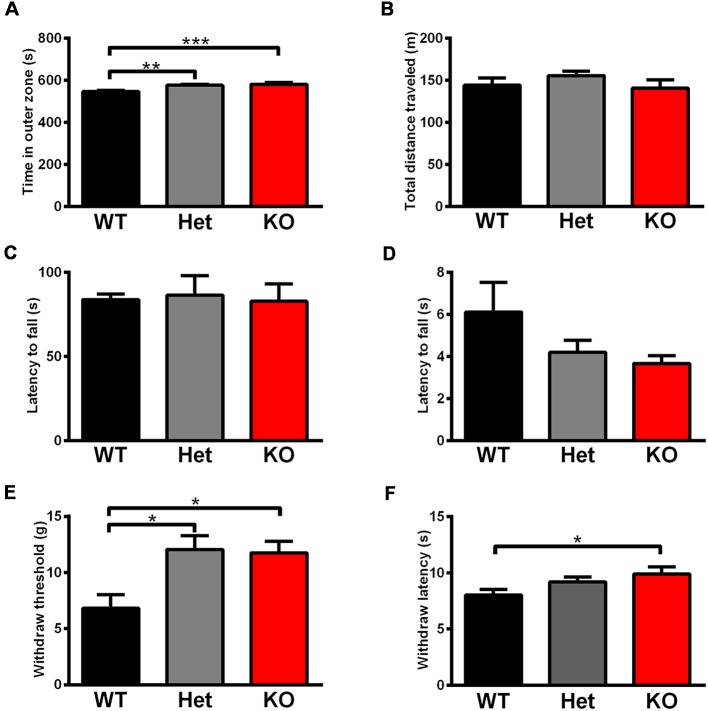
*Shank3*-deficient rats exhibited increased anxiety behavior and an increased pain threshold. **(A)** The amount of time spent close to the chamber walls in the open field test (WT, *n* = 13; Het, *n* = 18; KO, *n* = 10; Kruskal-Wallis test with Dunn’s multiple comparisons test). **(B)** Total distance traveled in the open field test (WT, *n* = 13; Het, *n* = 18; KO, *n* = 10; Kruskal-Wallis test). **(C)** The latency to fall from the rotarod in the rotarod test (WT, *n* = 18; Het, *n* = 8; KO, *n* = 11; one-way ANOVA). **(D)** The latency to fall in the hang wire test (WT, *n* = 12; Het, *n* = 7; KO, *n* = 12; Kruskal-Wallis test). **(E)** The withdrawal threshold in the von Frey test for punctate mechanical pain sensitivity (WT, *n* = 13; Het, *n* = 11; KO, *n* = 13; Kruskal-Wallis test with Dunn’s multiple comparisons test). **(F)** The withdrawal latency in the hot plate test for heat sensitivity at 53°C (WT, *n* = 23; Het, *n* = 18; KO, *n* = 19; Kruskal-Wallis test with Dunn’s multiple comparisons test). Data are presented as the mean ± SEM. *P < 0.05; **P < 0.01; ***P < 0.001.

Rats were tested in an open field to analyze their total activity and anxiety-like behavior. *Shank3* heterozygous and knockout rats exhibited increased anxiety behavior, as indicated by the increased amount of time spent in the outer region of the open field compared with wild-type littermates (*Shank3*^+/+^: 546.4 ± 6.498 s, *n* = 13; *Shank3*^+/–^: 576.8 ± 3.456 s, *n* = 18; *Shank3*^−/−^: 581.4 ± 9.220 s, *n* = 10; *P* < 0.001; Figure 5A). The total distances traveled in the open field did not differ between the genotypes (Figure 5B), which indicated normal activity in *Shank3*-deficient rats.

Since increased pain threshold and hypotonia have been reported in patients with *Shank3* mutations as frequent comorbidities, we conducted relevant behavior tests. The rotarod test showed that there were no differences in the latency to fall between the different genotypes (Figure 5C), which indicated normal motor coordination in *Shank3*-deficient rats. Neuromuscular strength and equilibrium were analyzed in the hang wire test, and *Shank3*-deficient rats exhibited no change compared to wild-type littermates (Figure 5D).

The von Frey test showed significant increases in paw withdrawal threshold to punctate mechanical stimuli of *Shank3* heterozygous and knockout rats (*Shank3*^+/+^: 6.812 ± 1.206 g, *n* = 13; *Shank3*^+/–^: 12.07 ± 1.229 g, *n* = 11; *Shank3*^−/−^: 11.76 ± 1.011 g, *n* = 13; *P* < 0.05; Figure 5E), indicating their increased mechanical pain threshold. The hot plate test was performed at 53°C, and the results showed that *Shank3* knockout rats displayed significantly increased paw withdrawal latency (*Shank3*^+/+^: 8.025 ± 0.5168 s, *n* = 23; *Shank3*^+/–^: 9.200 ± 0.4393 s, *n* = 18; *Shank3*^−/−^: 9.893 ± 0.6453 s, *n* = 19; *P* < 0.05; Figure 5F), which suggested a reduction in temperature sensation.

### Self-Grooming Behavior and Skin Lesions in *Shank3*-Deficient Rats

**Figure 6:**
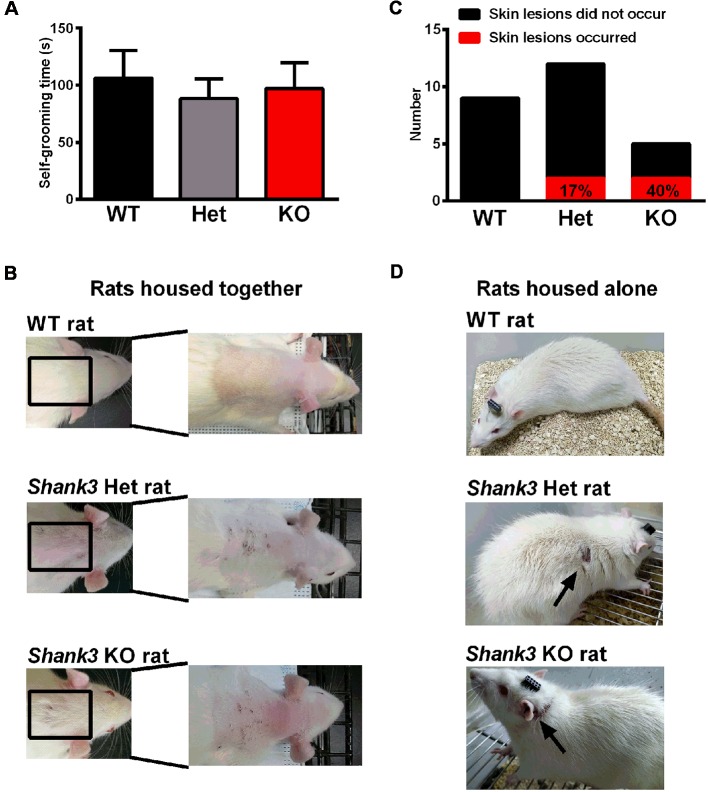
Self-grooming behavior and skin lesions in *Shank3*-deficient rats. **(A)** The amount of time spent in self-grooming in the self-grooming behavior test (WT, *n* = 12; Het, *n* = 13; KO, *n* = 11; Kruskal-Wallis test). Data are presented as the mean ± SEM. **(B)** Representative images of wild-type rats and skin lesions on the back of the neck in a few of *Shank3*-deficient rats that were housed with 3–5 total rats per cage. **(C)** The number of skin lesions that occurred or did not occur in rats that were housed alone. **(D)** Representative photographs of wild-type rats and skin lesions in *Shank3*-deficient rats that were housed alone.

Furthermore, repetitive behavior was analyzed in the self-grooming behavior test and the results showed that there was no genotype difference (Figure 6A), which only suggested that the self-grooming behaviors of 5- to 6-week-old *Shank3*-deficient rats were normal in this 10-min test. However, we observed skin lesions on the back of the neck in a few of the *Shank3*-deficient rats housed together that are not entirely explained at the moment (Figure 6B). Notably, the skin lesions were also observed in *Shank3*-deficient rats housed alone (Figures 6C,D), which was speculated to be related to excessive self-grooming behaviors or self-injurious behaviors.

### Oxytocin (OXT) and Arginine-Vasopressin (AVP) System Analysis

**Figure 7:**
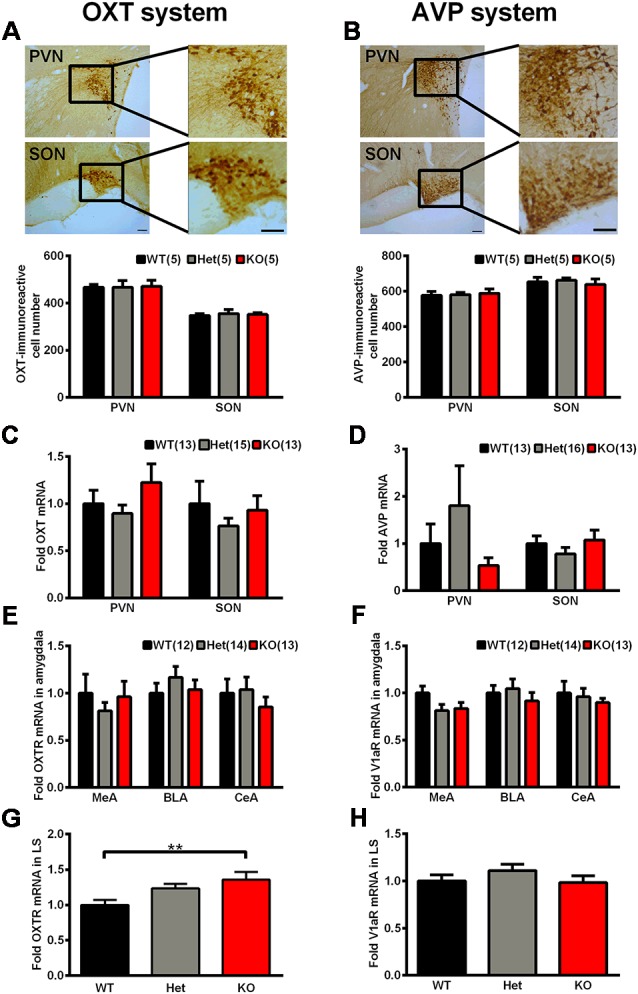
The oxytocin (OXT)/arginine-vasopressin (AVP) system in *Shank3*-deficient rats. **(A)** Representative photomicrograph of OXT-positive cells in the paraventricular nucleus (PVN) and supraoptic nucleus (SON; scale bar: 100 μm) and quantification of the number of OXT-immunoreactive cells in the PVN and SON (*n* = 5 rats for each genotype, six slices per rat; one-way ANOVA). **(B)** Representative photomicrograph of AVP-positive cells in the PVN and SON (scale bar: 100 μm) and quantification of the number of AVP-immunoreactive cells in the PVN and SON (*n* = 5 rats for each genotype, six slices per rat; one-way ANOVA). **(C)** mRNA expression levels of OXT in the PVN and SON (WT, *n* = 13–14; Het, *n* = 15–17; KO, *n* = 13–15; one-way ANOVA and Kruskal-Wallis test). **(D)** mRNA expression levels of AVP in the PVN and SON (WT, *n* = 13–14; Het, *n* = 16–17; KO, *n* = 12–15; Kruskal-Wallis test). **(E)** mRNA expression levels of OXTR in three subregions of the amygdala (MeA, BLA, and CeA; WT, *n* = 12; Het, *n* = 14; KO, *n* = 13; one-way ANOVA and Kruskal-Wallis test). **(F)** mRNA expression levels of V1aR in three subregions of the amygdala (MeA, BLA, and CeA; WT, *n* = 12; Het, *n* = 14; KO, *n* = 13; one-way ANOVA). **(G)** mRNA expression levels of OXTR in the lateral septum (LS; WT, *n* = 13; Het, *n* = 15; KO, *n* = 13; one-way ANOVA with Dunnett’s multiple comparisons test). **(H)** mRNA expression levels of V1aR in the LS (WT, *n* = 13; Het, *n* = 15; KO, *n* = 13; one-way ANOVA). Data are presented as the mean ± SEM. **P < 0.01.

As mentioned above, OXT and AVP are considered as social factors that are mainly synthesized in the PVN and SON and have been speculated to be related to mammalian social behavior and social memory. We thoroughly analyzed the OXT and AVP system in *Shank3*-deficient rats. We observed no significant genotype differences in the number of OXT-immunoreactive cells and AVP-immunoreactive cells in the PVN and SON (Figures 7A,B). Similarly, we also observed no significant differences in the mRNA expression levels of OXT and AVP in the PVN and SON between *Shank3*-deficient rats and their wild-type littermates (Figures 7C,D).

In mammals, there are three different receptors for AVP, including V1a, V1b, and V2; among them, the V1a receptor is the predominant form in the brain (Harony and Wagner, 2010; Manning et al., 2012). The amygdala and LS are two brain nuclei that express OXTR and V1aR and mediate the central actions of OXT and AVP on social behaviors and social memory (Lukas et al., 2013). Therefore, we analyzed OXTR and V1aR expression levels in these two nuclei. In three subregions of the amygdala, including the basolateral nucleus of the amygdala (BLA), the central nucleus of the amygdala (CeA) and the medial nucleus of the amygdala (MeA), the mRNA expression levels of OXTR and V1aR did not differ between the genotypes (Figures 7E,F). However, in the LS, we observed significantly increased OXTR mRNA expression levels (135.8 ± 11.04% of *Shank3*^+/+^, *n* = 13–15 per genotype, *P* < 0.01) and unchanged V1aR mRNA expression levels in *Shank3* knockout rats (Figures 7G,H).

## Discussion

*Shank3* mutations contribute to the genetic etiology of ASDs and *Shank3* is the major causative gene in the heterozygous deletion of the long arm of chromosome 22q, called PMS. In our present study, we established and thoroughly characterized a novel rat model with a complete *Shank3* deletion. Since alternative splicing of the *Shank3* gene has been reported (Wang et al., 2011), we confirmed that the full-length SHANK3 protein and major SHANK3 isoforms were disrupted completely in knockout rats using different SHANK3 antibodies, which is in contrast with other isoform-specific mutant mouse models and the rat model with a local deletion of exon 6 (Bozdagi et al., 2010; Peca et al., 2011; Wang et al., 2011; Schmeisser et al., 2012; Lee et al., 2015). This novel rat model with a complete *Shank3* deletion can likely better mimic the majority of patients carrying deletions of the entire *Shank3* gene in PMS (Soorya et al., 2013).

A series of biochemical analyses were also performed to examine whether SHANK family proteins and SHANK3-interacting proteins are altered in *Shank3* knockout rats. We did not find changes in other SHANK family proteins, which suggests that there no potential compensatory mechanisms related to other SHANK family proteins. The expression levels of other scaffolding proteins and glutamatergic receptors, including PSD-95, Homer, GluR1 and NR1, were altered, especially in the striatum. The striatum, which mediates components of rodent social behaviors and is related to parts of cognitive and sensorimotor abilities (Brielmaier et al., 2012), has previously been identified as one of the dysfunctional brain regions implicated in autism. In particular, it has been confirmed that the striatum is involved in repetitive behaviors in both autistic patients and animal models (Peca et al., 2011; Langen et al., 2014; Rothwell et al., 2014). Moreover, SHANK3 is the most abundant SHANK family member expressed in the striatum but not in other brain regions (Peca et al., 2011), which might explain why *Shank3* deficiency has a greater impact on the striatum. The altered molecular composition of *Shank3* knockout synapses confirmed the important role of SHANK3 as a scaffolding protein at PSDs. Compared to other *Shank3*-deficient animal models carrying different mutations, changes in protein expression levels in PSDs showed not only brain region-specific but also mutation-specific alterations, indicating complex biochemical mechanisms underlying individual *Shank3* mutations. Notably, we observed significant alterations of PSD95 and NMDAR1 subunits in *Shank3* heterozygous rats. This result could indicate that any alteration of *Shank3* levels (50% reduction in this study) might modulate the composition of PSDs. This is especially intriguing since human *Shank3* mutations are solely heterozygous disease entities. However, the molecular mechanism explaining these changes is far from being fully understood.

At glutamatergic synapses, SHANK3 interacts with multiple glutamate receptor subunits and the actin-based cytoskeleton as a scaffolding protein, and it is therefore critical for synaptic morphology and synaptic transmission. Normal dendritic spines contribute significantly to regular synaptic function and efficient neural circuit wiring, while altered dendritic spines often occur in individuals with autism-related disorders (Hutsler and Zhang, 2010; Tang et al., 2014), and in experimental mouse models, including some *Shank3*-deficient mouse strains (Peca et al., 2011; Wang et al., 2011, 2017). In the present study, we also observed decreased spine density in *Shank3* knockout rats, suggesting impaired spine formation, which may lead to impaired synaptic function and plasticity eventually. To this end, we analyzed synaptic function and plasticity, especially in the hippocampus. Basal synaptic transmission and LTP were both reduced in *Shank3* knockout rats. Therefore, we confirmed the effect of the *Shank3* mutation on glutamatergic synaptic function and plasticity. Impaired learning and memory formation, as well as impaired social memory, might also be consistent with dysfunctions in synaptic function and plasticity in the hippocampal formation.

Prompted by the crucial role of *Shank3* in synaptic function and the genetic etiology of ASDs and PMS, many *Shank3*-deficient mouse models carrying different mutations have been established. A spectrum of behavioral phenotypes, including impaired social behaviors and increased repetitive behavior, which are regarded as core symptoms of ASDs, have been described. Moreover, impaired learning and memory formation as well as increased anxiety behavior as comorbidities, have been identified (Bozdagi et al., 2010; Bangash et al., 2011; Peca et al., 2011; Wang et al., 2011; Yang et al., 2012; Kouser et al., 2013; Duffney et al., 2015). Subsequently, a rat model with a local deletion of exon 6 of *Shank3* was established, and normal social behavior but impaired social memory and attention were observed (Harony-Nicolas et al., 2017). Another *Shank3*-deficient nonhuman primate model with altered neurogenesis has also been reported (Zhao et al., 2017). In our present study, we did not find aberrant social interaction behaviors in *Shank3*-deficient rats, which have been identified in a majority of *Shank3*-deficient mouse models. These discrepancies may be attributed to species-specific differences, different genetic backgrounds, different targeting sites in the *Shank3* gene, different ages of animals used in behavioral tests and different paradigms of behavioral tests. Heterogeneity in clinical symptoms among patients with PMS and ASD and normal social behaviors performed by some of the patients with PMS should also be considered (Scheeren et al., 2012; Soorya et al., 2013). The normal levels of OXT and AVP in the hypothalamus and the normal levels of OXTR and V1aR in the amygdala might be one of the reasons for normal social behavior. To the best of our knowledge, the endogenous OXT/AVP system was explored for the first time in a *Shank3*-deficient animal model. Nevertheless, similar to different behavioral defects in *Shank3*-deficient animal models, we speculated that changes in the OXT/AVP system can also vary among different models and contribute to phenotypes related to social interaction. These results also suggest that there are independent pathways responding to social novelty recognition and social interaction ability, even in the same gene knockout condition.

In this genetically modified rat model, we observed impaired social memory indicated by deficits in social novelty recognition. Olfaction plays an important role in social recognition in rodents (Wacker and Ludwig, 2012), and social recognition tests make use of the natural tendency of animals toward the olfactory investigation of novel conspecifics (Bielsky and Young, 2004). Typically, OHDTs are performed as a control task. Impaired social behavior with normal olfactory ability has also been reported in *Shank3*-deficient mice with a deletion of exons 4 through 9 (Bozdagi et al., 2010). In this study, normal olfactory ability suggested that dysfunctions in other complex and elaborate mechanisms of processing social stimuli and memory led to impaired social memory in *Shank3*-deficient rats. OXT and AVP also play an important role in social memory, which can act on a variety of brain regions to facilitate social recognition and modulate subsequent behavioral responses (Wacker and Ludwig, 2012). Among these, the LS is thought to be crucial for the mediation of social recognition (van der Kooij and Sandi, 2012). However, analysis of OXTR and V1aR expression levels in the LS showed increased mRNA expression levels of OXTR even with normal synthetic levels of OXT. Currently, we cannot explain exactly the impaired social memory and the divergent changes of the OXT/AVP system. The involvement of acetylcholine, norepinephrine, dopamine, serotonin and estrogen in social memory has also been reported (van der Kooij and Sandi, 2012; Ervin et al., 2015). In addition, secretin can increase the acquisition of social recognition in an OXTR-dependent manner (Takayanagi et al., 2017). Therefore, we doubt that there are any cross-mediating mechanisms. The various neurotransmitter systems committed to social memory and the interactions between other neurotransmitters and the OXT/AVP system should be further analyzed. Notably, the facilitatory effect of OXT administration on a variety of social behaviors has been reported not only in humans (Domes et al., 2007; Guastella et al., 2008; Hurlemann et al., 2010) but also in rodents (Lukas et al., 2011). In particular, the improved effect of OXT treatment on social memory and attention has been observed in a *Shank3*-deficient rat model with a deletion in exon 6 (Harony-Nicolas et al., 2017). Combining these results from studies of humans and animals with normal or impaired social behavior, we can infer that there is an ameliorative effect of OXT on social memory even with a normal OXT/AVP system. Therefore, whether the OXT treatment can improve the defective behaviors in this *Shank3*-deficient rat should also be further studied.

Cognitive disabilities, anxiety, sensory-perceptual anomalies and pain insensitivity are common comorbidities in patients with ASD and PMS (Allely, 2013; Soorya et al., 2013; Croen et al., 2015; Polyak et al., 2015). Analogously, we observed impaired learning and memory, increased anxiety behavior, an increased baseline mechanical pain threshold and reduced temperature sensation in *Shank3*-deficient rats. Impaired learning and memory and increased anxiety behavior have often been reported in a majority of *Shank3*-deficient mouse models (Bozdagi et al., 2010; Bangash et al., 2011; Peca et al., 2011; Wang et al., 2011; Yang et al., 2012; Kouser et al., 2013; Duffney et al., 2015). Impaired heat hyperalgesia in inflammatory and neuropathic pain and the interaction between SHANK3 and transient receptor potential subtype V1 (TRPV1) have been reported in *Shank3* knockout mice with a deletion of exons 4–22, however, there was no change in the baseline thermal and mechanical sensitivity in this mouse model as we observed (Han et al., 2016). Incorporating these findings, *Shank3* mutation may underlie pain deficits in *Shank3*-related ASD and PMS through some mechanisms that include the interaction with TRPV1 but are not limited to.

Notably, to understand the results of the behavioral tests in our present study, limitations of some behavioral tests for rats should also be considered. In particular, the detection of muscle and motor function using other methods and the observation of self-grooming behavior in home cages should be further performed.

Overall, the *Shank3*-deficient rat model established in this study showed impaired synaptic function as expected, and a spectrum of behavioral phenotypes related to comorbidities in PMS and ASDs, which make it possible to assess potential therapeutics for PMS, ASDs and other developmental disorders using this model.

## Data Availability

The datasets generated for this study are available on request to the corresponding author.

## Author Contributions

T-JS conceived and designed the study and wrote the manuscript. T-JS, X-YL and M-PW performed the experiments and analyzed the data. F-JZ, J-NW, SY, M-YJ, Y-FX, W-WD, P-WS and Y-YS helped to perform the experiments. RZ, J-SH, and S-PH helped to design the study and contributed to analysis with constructive discussions. RZ, TB, MS, CZ and M-HQ reviewed and edited the manuscript and made valuable suggestions. All authors approved the final version.

## Conflict of Interest Statement

S-PH was employed by company Wuxi HANS Health Medical Technology Co., 725 Ltd. The remaining authors declare that the research was conducted in the absence of any commercial or financial relationships that could be construed as a potential conflict of interest.

## Footnotes

^1^http://crispr.mit.edu

## References

[B1] Allely C. S. (2013). Pain sensitivity and observer perception of pain in individuals with autistic spectrum disorder. ScientificWorldJournal 2013:916178. 10.1155/2013/916178 23843740 PMC3697411

[B2] Bangash M. A. Park J. M. Melnikova T. Wang D. Jeon S. K. Lee D.. (2011). Enhanced polyubiquitination of Shank3 and NMDA receptor in a mouse model of autism. Cell 145, 758–772. 10.1016/j.cell.2011.03.052 21565394 PMC3110672

[B3] Baron M. K. Boeckers T. M. Vaida B. Faham S. Gingery M. Sawaya M. R.. (2006). An architectural framework that may lie at the core of the postsynaptic density. Science 311, 531–535. 10.1126/science.1118995 16439662

[B4] Bian W. J. Miao W. Y. He S. J. Qiu Z. Yu X. (2015). Coordinated spine pruning and maturation mediated by inter-spine competition for cadherin/catenin complexes. Cell 162, 808–822. 10.1016/j.cell.2015.07.018 26255771

[B5] Bielsky I. F. Young L. J. (2004). Oxytocin, vasopressin and social recognition in mammals. Peptides 25, 1565–1574. 10.1016/j.peptides.2004.05.019 15374658

[B6] Boeckers T. M. (2006). The postsynaptic density. Cell Tissue Res. 326, 409–422. 10.1007/s00441-006-0274-5 16865346

[B7] Boeckers T. M. Winter C. Smalla K. H. Kreutz M. R. Bockmann J. Seidenbecher C.. (1999). Proline-rich synapse-associated proteins ProSAP1 and ProSAP2 interact with synaptic proteins of the SAPAP/GKAP family. Biochem. Biophys. Res. Commun. 264, 247–252. 10.1006/bbrc.1999.1489 10527873

[B8] Bozdagi O. Sakurai T. Papapetrou D. Wang X. Dickstein D. L. Takahashi N.. (2010). Haploinsufficiency of the autism-associated Shank3 gene leads to deficits in synaptic function, social interaction, and social communication. Mol. Autism 1:15. 10.1186/2040-2392-1-15 21167025 PMC3019144

[B9] Brielmaier J. Matteson P. G. Silverman J. L. Senerth J. M. Kelly S. Genestine M.. (2012). Autism-relevant social abnormalities and cognitive deficits in engrailed-2 knockout mice. PLoS One 7:e40914. 10.1371/journal.pone.0040914 22829897 PMC3400671

[B10] Croen L. A. Zerbo O. Qian Y. Massolo M. L. Rich S. Sidney S.. (2015). The health status of adults on the autism spectrum. Autism 19, 814–823. 10.1177/1362361315577517 25911091

[B11] Domes G. Heinrichs M. Michel A. Berger C. Herpertz S. C. (2007). Oxytocin improves “mind-reading” in humans. Biol. Psychiatry 61, 731–733. 10.1016/j.biopsych.2006.07.015 17137561

[B12] Duffney L. J. Zhong P. Wei J. Matas E. Cheng J. Qin L.. (2015). Autism-like deficits in shank3-deficient mice are rescued by targeting actin regulators. Cell Rep. 11, 1400–1413. 10.1016/j.celrep.2015.04.064 26027926 PMC4464902

[B13] Ervin K. S. Lymer J. M. Matta R. Clipperton-Allen A. E. Kavaliers M. Choleris E. (2015). Estrogen involvement in social behavior in rodents: rapid and long-term actions. Horm. Behav. 74, 53–76. 10.1016/j.yhbeh.2015.05.023 26122289

[B14] Gigliucci V. Leonzino M. Busnelli M. Luchetti A. Palladino V. S. D’Amato F. R.. (2014). Region specific up-regulation of oxytocin receptors in the opioid oprm1^−/−^ mouse model of autism. Front. Pediatr. 2:91. 10.3389/fped.2014.00091 25225634 PMC4150055

[B15] Gong X. Jiang Y. W. Zhang X. An Y. Zhang J. Wu Y.. (2012). High proportion of 22q13 deletions and SHANK3 mutations in Chinese patients with intellectual disability. PLoS One 7:e34739. 10.1371/journal.pone.0034739 22509352 PMC3324537

[B16] Grabrucker A. M. Schmeisser M. J. Schoen M. Boeckers T. M. (2011). Postsynaptic ProSAP/Shank scaffolds in the cross-hair of synaptopathies. Trends Cell Biol. 21, 594–603. 10.1016/j.tcb.2011.07.003 21840719

[B17] Gray E. G. (1959). Electron microscopy of synaptic contacts on dendrite spines of the cerebral cortex. Nature 183, 1592–1593. 10.1038/1831592a0 13666826

[B18] Guastella A. J. Mitchell P. B. Dadds M. R. (2008). Oxytocin increases gaze to the eye region of human faces. Biol. Psychiatry 63, 3–5. 10.1016/j.biopsych.2007.06.026 17888410

[B19] Han Q. Kim Y. H. Wang X. Liu D. Zhang Z. J. Bey A. L.. (2016). SHANK3 deficiency impairs heat hyperalgesia and TRPV1 signaling in primary sensory neurons. Neuron 92, 1279–1293. 10.1016/j.neuron.2016.11.007 27916453 PMC5182147

[B20] Harony H. Wagner S. (2010). The contribution of oxytocin and vasopressin to mammalian social behavior: potential role in autism spectrum disorder. Neurosignals 18, 82–97. 10.1159/000321035 21150165

[B21] Harony-Nicolas H. Kay M. Hoffmann J. D. Klein M. E. Bozdagi-Gunal O. Riad M.. (2017). Oxytocin improves behavioral and electrophysiological deficits in a novel Shank3-deficient rat. Elife 6:e18904. 10.7554/elife.18904 28139198 PMC5283828

[B22] Hurlemann R. Patin A. Onur O. A. Cohen M. X. Baumgartner T. Metzler S.. (2010). Oxytocin enhances amygdala-dependent, socially reinforced learning and emotional empathy in humans. J. Neurosci. 30, 4999–5007. 10.1523/JNEUROSCI.5538-09.2010 20371820 PMC6632777

[B23] Hutsler J. J. Zhang H. (2010). Increased dendritic spine densities on cortical projection neurons in autism spectrum disorders. Brain Res. 1309, 83–94. 10.1016/j.brainres.2009.09.120 19896929

[B24] Ishikawa H. Yamada K. Pavlides C. Ichitani Y. (2014). Sleep deprivation impairs spontaneous object-place but not novel-object recognition in rats. Neurosci. Lett. 580, 114–118. 10.1016/j.neulet.2014.08.004 25123440

[B25] Janssen E. Göhlen B. Behrens D. Richter K. Zavazava N. (2001). Allogeneic recombinant soluble MHC class I molecules modify urinary odor cues in rats. Physiol. Behav. 72, 107–114. 10.1016/s0031-9384(00)00389-9 11239987

[B26] Jin D. Liu H. X. Hirai H. Torashima T. Nagai T. Lopatina O.. (2007). CD38 is critical for social behaviour by regulating oxytocin secretion. Nature 446, 41–45. 10.1038/nature05526 17287729

[B27] Kouser M. Speed H. E. Dewey C. M. Reimers J. M. Widman A. J. Gupta N.. (2013). Loss of predominant Shank3 isoforms results in hippocampus-dependent impairments in behavior and synaptic transmission. J. Neurosci. 33, 18448–18468. 10.1523/JNEUROSCI.3017-13.2013 24259569 PMC3834052

[B28] Langen M. Bos D. Noordermeer S. D. Nederveen H. van Engeland H. Durston S. (2014). Changes in the development of striatum are involved in repetitive behavior in autism. Biol. Psychiatry 76, 405–411. 10.1016/j.biopsych.2013.08.013 24090791

[B29] Leblond C. S. Nava C. Polge A. Gauthier J. Huguet G. Lumbroso S.. (2014). Meta-analysis of SHANK mutations in autism spectrum disorders: a gradient of severity in cognitive impairments. PLoS Genet. 10:e1004580. 10.1371/journal.pgen.1004580 25188300 PMC4154644

[B30] Lee J. Chung C. Ha S. Lee D. Kim D. Y. Kim H.. (2015). Shank3-mutant mice lacking exon 9 show altered excitation/inhibition balance, enhanced rearing, and spatial memory deficit. Front. Cell. Neurosci. 9:94. 10.3389/fncel.2015.00094 25852484 PMC4365696

[B31] Li J. Chai A. Wang L. Ma Y. Wu Z. Yu H.. (2015). Synaptic P-Rex1 signaling regulates hippocampal long-term depression and autism-like social behavior. Proc. Natl. Acad. Sci. U S A 112, E6964–E6972. 10.1073/pnas.1512913112 26621702 PMC4687539

[B32] Li M. He W. Heupel K. (2011). Administration of clozapine to a mother rat potentiates pup ultrasonic vocalization in response to separation and re-separation: contrast with haloperidol. Behav. Brain Res. 222, 385–389. 10.1016/j.bbr.2011.03.062 21473887 PMC3096738

[B33] Lukas M. Toth I. Reber S. O. Slattery D. A. Veenema A. H. Neumann I. D. (2011). The neuropeptide oxytocin facilitates pro-social behavior and prevents social avoidance in rats and mice. Neuropsychopharmacology 36, 2159–2168. 10.1038/npp.2011.95 21677650 PMC3176581

[B34] Lukas M. Toth I. Veenema A. H. Neumann I. D. (2013). Oxytocin mediates rodent social memory within the lateral septum and the medial amygdala depending on the relevance of the social stimulus: male juvenile versus female adult conspecifics. Psychoneuroendocrinology 38, 916–926. 10.1016/j.psyneuen.2012.09.018 23102690

[B35] Manning M. Misicka A. Olma A. Bankowski K. Stoev S. Chini B.. (2012). Oxytocin and vasopressin agonists and antagonists as research tools and potential therapeutics. J Neuroendocrinol 24, 609–628. 10.1111/j.1365-2826.2012.02303.x 22375852 PMC3490377

[B36] Naisbitt S. Kim E. Tu J. C. Xiao B. Sala C. Valtschanoff J.. (1999). Shank, a novel family of postsynaptic density proteins that binds to the NMDA receptor/PSD-95/GKAP complex and cortactin. Neuron 23, 569–582. 10.1016/s0896-6273(00)80809-0 10433268

[B37] Peca J. Feliciano C. Ting J. T. Wang W. Wells M. F. Venkatraman T. N.. (2011). Shank3 mutant mice display autistic-like behaviours and striatal dysfunction. Nature 472, 437–442. 10.1038/nature09965 21423165 PMC3090611

[B38] Penagarikano O. Lazaro M. T. Lu X. H. Gordon A. Dong H. Lam H. A.. (2015). Exogenous and evoked oxytocin restores social behavior in the Cntnap2 mouse model of autism. Sci. Transl. Med. 7:271ra278. 10.1126/scitranslmed.3010257 25609168 PMC4498455

[B39] Polyak A. Kubina R. M. Girirajan S. (2015). Comorbidity of intellectual disability confounds ascertainment of autism: implications for genetic diagnosis. Am. J. Med. Genet. B Neuropsychiatr. Genet. 168, 600–608. 10.1002/ajmg.b.32338 26198689

[B40] Pyndt Jørgensen B. Krych L. Pedersen T. B. Plath N. Redrobe J. P. Hansen A. K.. (2015). Investigating the long-term effect of subchronic phencyclidine-treatment on novel object recognition and the association between the gut microbiota and behavior in the animal model of schizophrenia. Physiol. Behav. 141, 32–39. 10.1016/j.physbeh.2014.12.042 25545766

[B41] Rothwell P. E. Fuccillo M. V. Maxeiner S. Hayton S. J. Gokce O. Lim B. K.. (2014). Autism-associated neuroligin-3 mutations commonly impair striatal circuits to boost repetitive behaviors. Cell 158, 198–212. 10.1016/j.cell.2014.04.045 24995986 PMC4120877

[B42] Scheeren A. M. Koot H. M. Begeer S. (2012). Social interaction style of children and adolescents with high-functioning autism spectrum disorder. J. Autism Dev. Disord. 42, 2046–2055. 10.1007/s10803-012-1451-x 22294525

[B43] Schmeisser M. J. Ey E. Wegener S. Bockmann J. Stempel A. V. Kuebler A.. (2012). Autistic-like behaviours and hyperactivity in mice lacking ProSAP1/Shank2. Nature 486, 256–260. 10.1038/nature11015 22699619

[B44] Soorya L. Kolevzon A. Zweifach J. Lim T. Dobry Y. Schwartz L.. (2013). Prospective investigation of autism and genotype-phenotype correlations in 22q13 deletion syndrome and SHANK3 deficiency. Mol. Autism 4:18. 10.1186/2040-2392-4-18 23758760 PMC3707861

[B45] Takayanagi Y. Yoshida M. Takashima A. Takanami K. Yoshida S. Nishimori K.. (2017). Activation of supraoptic oxytocin neurons by secretin facilitates social recognition. Biol. Psychiatry 81, 243–251. 10.1016/j.biopsych.2015.11.021 26803341

[B46] Tang G. Gudsnuk K. Kuo S. H. Cotrina M. L. Rosoklija G. Sosunov A.. (2014). Loss of mTOR-dependent macroautophagy causes autistic-like synaptic pruning deficits. Neuron 83, 1131–1143. 10.1016/j.neuron.2014.07.040 25155956 PMC4159743

[B47] van der Kooij M. A. Sandi C. (2012). Social memories in rodents: methods, mechanisms and modulation by stress. Neurosci. Biobehav. Rev. 36, 1763–1772. 10.1016/j.neubiorev.2011.10.006 22079398

[B48] Vanderschuren L. J. Stein E. A. Wiegant V. M. Van Ree J. M. (1995a). Social isolation and social interaction alter regional brain opioid receptor binding in rats. Eur. Neuropsychopharmacol. 5, 119–127. 10.1016/0924-977x(95)00010-m 7549454

[B49] Vanderschuren L. J. Stein E. A. Wiegant V. M. Van Ree J. M. (1995b). Social play alters regional brain opioid receptor binding in juvenile rats. Brain Res. 680, 148–156. 10.1016/0006-8993(95)00256-p 7663971

[B50] Wacker D. W. Ludwig M. (2012). Vasopressin, oxytocin, and social odor recognition. Horm. Behav. 61, 259–265. 10.1016/j.yhbeh.2011.08.014 21920364

[B52] Wang X. Bey A. L. Katz B. M. Badea A. Kim N. David L. K.. (2016). Altered mGluR5-Homer scaffolds and corticostriatal connectivity in a Shank3 complete knockout model of autism. Nat. Commun. 7:11459. 10.1038/ncomms11459 27161151 PMC4866051

[B51] Wang W. Li C. Chen Q. van der Goes M. S. Hawrot J. Yao A. Y.. (2017). Striatopallidal dysfunction underlies repetitive behavior in Shank3-deficient model of autism. J. Clin. Invest. 127, 1978–1990. 10.1172/jci87997 28414301 PMC5409790

[B53] Wang X. McCoy P. A. Rodriguiz R. M. Pan Y. Je H. S. Roberts A. C.. (2011). Synaptic dysfunction and abnormal behaviors in mice lacking major isoforms of Shank3. Hum. Mol. Genet. 20, 3093–3108. 10.1093/hmg/ddr212 21558424 PMC3131048

[B54] Wei W. Song Y. Fan X. Zhang S. Wang L. Xu S.. (2016). Simultaneous recording of brain extracellular glucose, spike and local field potential in real time using an implantable microelectrode array with nano-materials. Nanotechnology 27:114001. 10.1088/0957-4484/27/11/114001 26871752

[B55] Wilson H. L. Wong A. C. Shaw S. R. Tse W. Y. Stapleton G. A. Phelan M. C.. (2003). Molecular characterisation of the 22q13 deletion syndrome supports the role of haploinsufficiency of SHANK3/PROSAP2 in the major neurological symptoms. J. Med. Genet. 40, 575–584. 10.1136/jmg.40.8.575 12920066 PMC1735560

[B56] Xu X. J. Zhang H. F. Shou X. J. Li J. Jing W. L. Zhou Y.. (2015). Prenatal hyperandrogenic environment induced autistic-like behavior in rat offspring. Physiol. Behav. 138, 13–20. 10.1016/j.physbeh.2014.09.014 25455866

[B58] Yang M. Bozdagi O. Scattoni M. L. Wöhr M. Roullet F. I. Katz A. M.. (2012). Reduced excitatory neurotransmission and mild autism-relevant phenotypes in adolescent Shank3 null mutant mice. J. Neurosci. 32, 6525–6541. 10.1523/JNEUROSCI.6107-11.2012 22573675 PMC3362928

[B57] Yang H. Wang H. Shivalila C. S. Cheng A. W. Shi L. Jaenisch R. (2013). One-step generation of mice carrying reporter and conditional alleles by CRISPR/Cas-mediated genome engineering. Cell 154, 1370–1379. 10.1016/j.cell.2013.08.022 23992847 PMC3961003

[B59] Zhang H. F. Dai Y. C. Wu J. Jia M. X. Zhang J. S. Shou X. J.. (2016). Plasma oxytocin and arginine-vasopressin levels in children with autism spectrum disorder in china: associations with symptoms. Neurosci. Bull. 32, 423–432. 10.1007/s12264-016-0046-5 27342432 PMC5563759

[B60] Zhang H. F. Li H. X. Dai Y. C. Xu X. J. Han S. P. Zhang R.. (2015). Electro-acupuncture improves the social interaction behavior of rats. Physiol. Behav. 151, 485–493. 10.1016/j.physbeh.2015.08.014 26265492

[B61] Zhang R. Zhang H. F. Han J. S. Han S. P. (2017). Genes related to oxytocin and arginine-vasopressin pathways: associations with autism spectrum disorders. Neurosci. Bull. 33, 238–246. 10.1007/s12264-017-0120-7 28283809 PMC5360847

[B62] Zhao H. Tu Z. Xu H. Yan S. Yan H. Zheng Y.. (2017). Altered neurogenesis and disrupted expression of synaptic proteins in prefrontal cortex of SHANK3-deficient non-human primate. Cell Res. 27, 1293–1297. 10.1038/cr.2017.95 28741620 PMC5630686

