# Supplementary material for: Altered Behaviors and Impaired Synaptic Function in a Novel Rat Model With a Complete Shank3 Deletion
Source: Front Cell Neurosci. 2019 Mar 26;13:111. doi: 10.3389/fncel.2019.00111 (PMC6444209; doi:10.3389/fncel.2019.00111)
Supplement: Supplementary file 1 [file Data_Sheet_1.PDF]

Supplementary Material

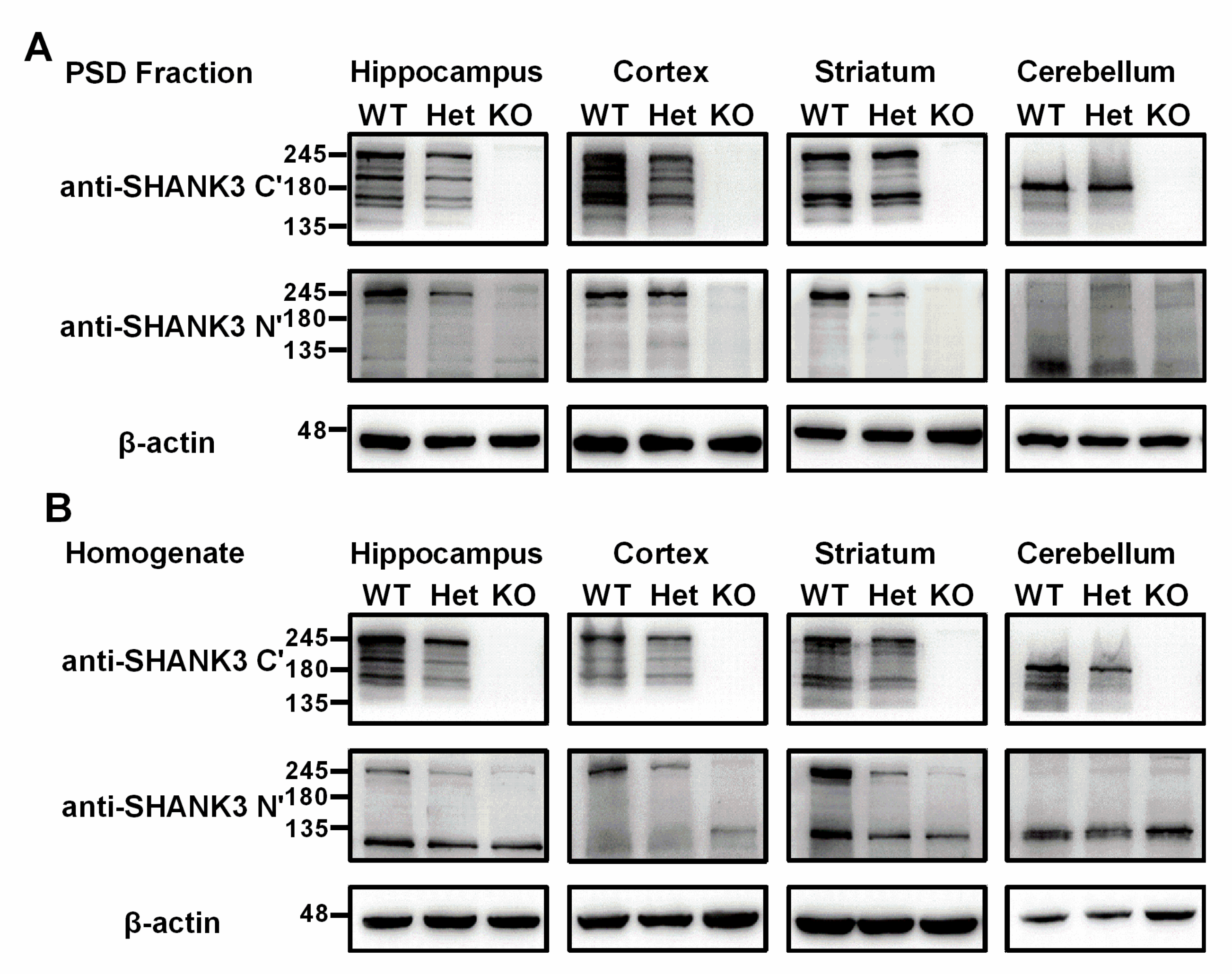

Supplementary Figure 1. Expression of SHANK3 in the PSD fractions and total homogenates from the hippocampus, cortex, striatum and cerebellum. **(A)** Representative immunoblots of the PSD fractions from the hippocampus, cortex, striatum and cerebellum probed with two anti-SHANK3 antibodies directed against the C-terminus or N-terminus. **(B)** Representative immunoblots of homogenates from the hippocampus, cortex, striatum and cerebellum probed with the two anti-SHANK3 antibodies.

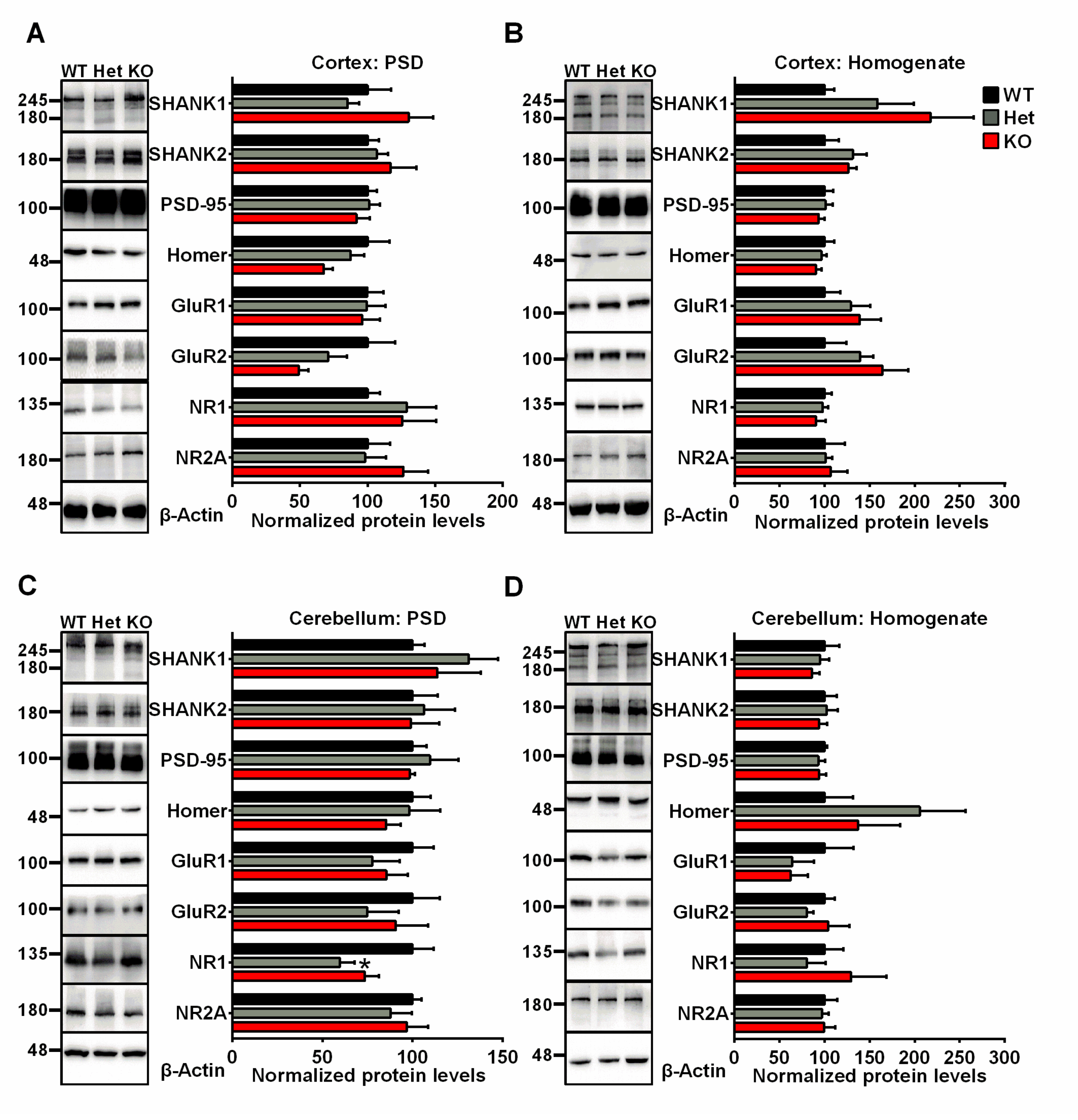

Supplementary Figure 2. Expression of synaptic proteins in PSD fractions and total homogenates from the cortex and cerebellum. **(A)** Representative immunoblots of PSD fractions from the cortex probed with different antibodies as indicated (left panel). Quantification of the corresponding protein expression levels normalized to β-actin (right panel) **(B)** Representative immunoblots of total homogenates from the cortex probed with different antibodies as indicated (left panel). Quantification of the corresponding protein expression levels normalized to β-actin (right panel). **(C)** Representative immunoblots of PSD fractions from the cerebellum probed with different antibodies as indicated (left panel). Quantification of the corresponding protein expression levels normalized to β-actin (right panel). **(D)** Representative immunoblots of total homogenates from the cerebellum probed with different antibodies as indicated (left panel). Quantification of the corresponding protein expression

levels normalized to β-actin (right panel). (WT, n=5; Het, n=5-6; KO, n=6; one-way ANOVA with Dunnett's multiple comparisons test). Data are presented as the mean ± SEM. *, $P < 0.05$; **, $P < 0.01$; ***, $P < 0.001$.

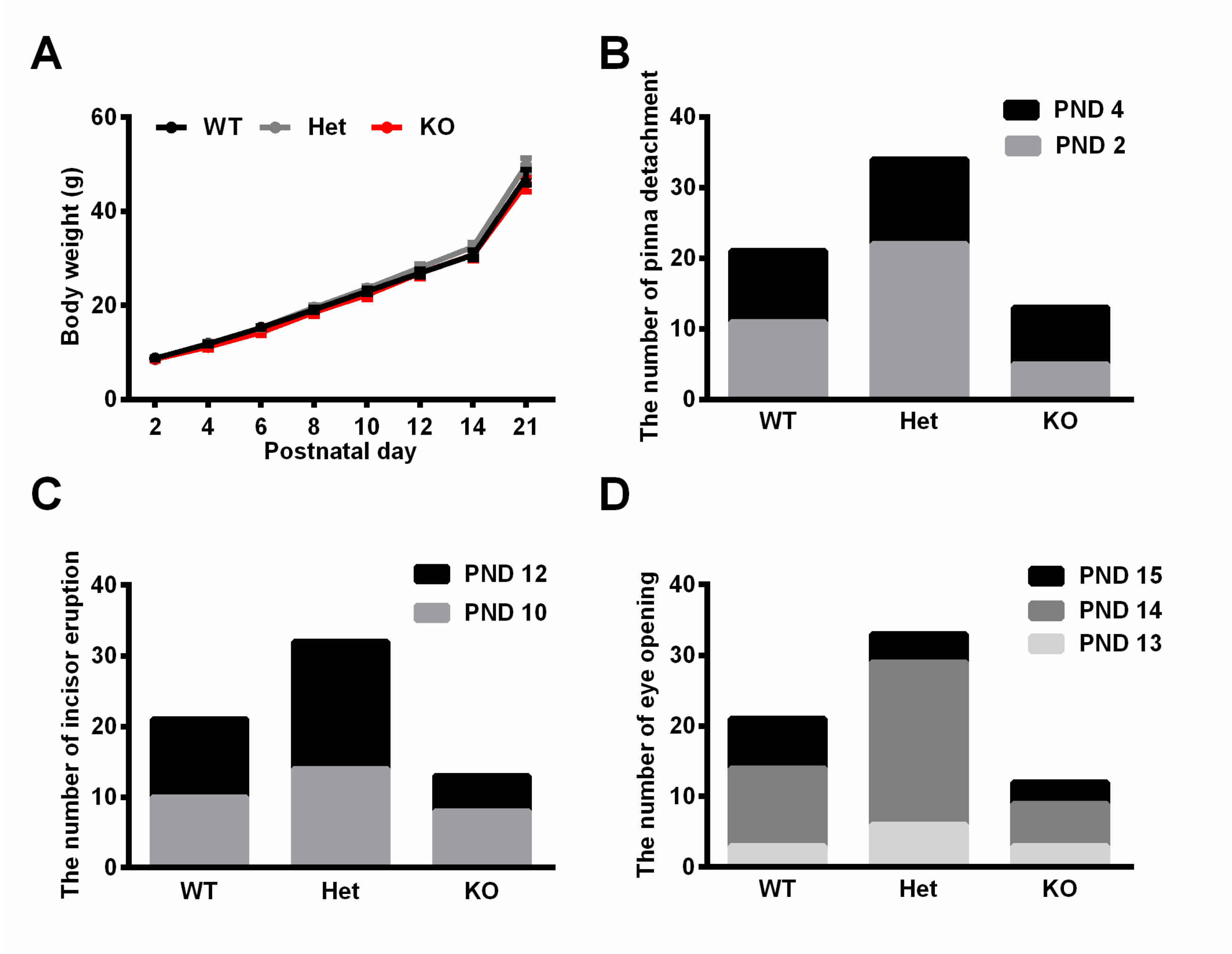

Supplementary Figure 3. Normal developmental milestones in *Shank3*-deficient rats. **(A)** Body weight of pups between PND2 and PND21. **(B)** The number of pups that finished pinna detachment on PND2 or PND4. **(C)** The number of pups that finished incisor eruption on PND10 or PND12. **(D)** The number of pups that finished eye opening on PND13 to PND15. (WT, n=21; Het, n=34; KO, n=13; two-way ANOVA, Pearson's chi-squared test).
